# A stochastic framework to assess the optimal allocation of limited vaccine doses in foot-and-mouth disease outbreaks using game theory

**DOI:** 10.3389/fvets.2026.1681056

**Published:** 2026-02-09

**Authors:** Karla I. Moreno-Torres, Michael W. Sanderson, Columb Rigney, Melissa Schoenbaum, Amy Delgado, Jonathan Arzt, Jessica L. Heier Stamm

**Affiliations:** 1 Department of Diagnostic Medicine/Pathobiology, Center for Outcomes Research and Epidemiology, Kansas State University, Manhattan, KS, United States; 2 United States Department of Agriculture, Animal and Plant Health Inspection Service, Center for Epidemiology and Animal Health, Fort Collins, CO, United States; 3 Foreign Animal Disease Research Unit, United States Department of Agriculture, Agricultural Research Service, Plum Island Animal Disease Center, Greenport, NY, United States; 4 Department of Industrial and Manufacturing Systems Engineering, Kansas State University, Manhattan, KS, United States

**Keywords:** decision-makers, FMD, foot-and-mouth disease, game theory, modeling, resource allocation, stochastic, vaccination

## Abstract

**Introduction:**

The necessary response to a livestock transboundary infectious disease outbreak will likely outpace available resources. Consequently, policymakers need strategies to inform decisions about allocating limited resources. This study aimed to develop a stochastic framework that strategically examines vaccine allocation in a series of simultaneous multi-player decision sets, using game theory and allocation rules.

**Methods:**

We modeled 11 stochastic foot-and-mouth disease (FMD) scenarios using InterSpread Plus (Version 6.01.44). Stakeholders were designated as decision-maker one (DM1, the index state) and decision-maker two (DM2, a group of three neighboring states) requesting all or a share of the available vaccines. We selected two outcome criteria for examination: outbreak size and duration. Vaccine allocation strategies were determined by four rules. Rule 1 prioritized allocation to the index state, rule 2 prioritized allocation to neighboring states, rule 3 provided equal prioritization, and rule 4 prioritized allocation based on the percentage of dairy cattle in each state. For each scenario, 300 iterations were completed using matched random seeds. The outcome rankings of each matched iteration were treated as the payoffs of DMs and were analyzed as static games with perfect information. Nash equilibrium and Pareto optimal solutions were summarized across scenarios and iterations within each rule. Decision positions were evaluated per allocation rule according to game theory equilibrium principles.

**Results:**

Rule 3, equal prioritization, resulted in a Pareto optimal solution more frequently, benefiting both DMs, and had the highest level of agreement in decision-states between Nash equilibrium and Pareto optimal outcomes. For rules 1–3, Pareto optimal solutions did not consistently result in lower outbreak size and duration (90th percentile) for both DMs when compared to Nash equilibrium solutions. Under rule 4, outbreak size and duration metrics showed less differentiation between decision positions.

**Discussion:**

This stochastic framework incorporates epidemiological data and accounts for the payoffs resulting from multiple stakeholders’ choices. This approach can aid in decision-making for scarce resource allocation in contexts where individual payoffs depend on others’ choices. Additionally, these findings contribute to improving preparedness for an outbreak of FMD in disease-free regions.

## 1 Introduction

Vaccination for livestock transboundary infectious diseases such as foot-and-mouth disease (FMD) in FMD-free countries has been increasingly considered in response to an outbreak as policymakers recognize that depopulation and carcass disposal may not be sufficiently available, adequate to address animal welfare concerns, or cost-effective for controlling an epidemic (1–3). As a result, in 1982, the North American Foot-and-Mouth Disease Vaccine Bank (NAFMDVB) was created to support vaccination strategies for FMD in North America. In 2010, the U. S. FMD Response Plan: Red Book was updated to clarify that the U. S. Department of Agriculture (USDA) Veterinary Services would consider using all vaccination strategies, including vaccinate-to-slaughter and vaccinate-to-live approaches, in the event of an outbreak of FMD (4). Additionally, in 2018, the United States expanded its vaccine stockpile by establishing the National Animal Vaccine and Veterinary Countermeasures Bank (National Bank) (4). The creation of the vaccine banks provides USDA and livestock producers with an additional tool to control FMD in the event of an incursion.

The initial vaccine availability from NAFMDVB and the National Bank is estimated to be approximately 2.5 million doses, arriving at a U. S. port 10–14 days after the placement of an order, assuming that a vaccine antigen concentrate appropriately matched with the FMD strain causing the epidemic is present within the stockpile (4). The United States National Agricultural Statistics Service (NASS) 2022 Agricultural Census estimated 91.9 million cattle and 72.2 million swine distributed throughout the U. S. Numerous individual states have populations with more than 2 million head of cattle or swine (5). Clearly, the number of FMD-susceptible animals far exceeds the initially available vaccine doses. The size and complexity of U. S. livestock production could make allocating initial vaccine doses a significant challenge when responding to an FMD outbreak.

Consequently, policymakers need strategies to inform decisions about allocating limited vaccine resources. Epidemiological models have a rich history of testing the efficacy of intervention strategies and are increasingly accepted and used in decision-making (6, 7). The majority of modeled vaccination strategies have advanced knowledge regarding one or more of the following issues: timing of vaccination (prophylactic or reactive), vaccination goal (protective or containment), spatial allocation (mass or ring vaccination), targeted population (cattle or swine, small farms or large farms), vaccine efficacy (time to immunity and levels of protection), social dimension aspect (stakeholders’ uptake of the vaccine), and logistics (shipment delays, vaccination capacity) (1, 2, 8–11). Nevertheless, few FMD modeling studies have incorporated optimal vaccine allocation. Tildesley et al. (12) developed an optimal reactive vaccination program based on the 2001 UK FMD epidemic. They investigated the optimal radius size that would minimize the epidemic impact in terms of the number of farms infected and culled. Results showed the optimal ring size given plausible logistical constraints, such as capacity, total vaccine doses, vaccine efficacies, and epidemic size before vaccination begins. In addition, the authors suggested that when new foci of infection arise, the optimal allocation of vaccination should prioritize the nearest farms to any infected premises and/or to dangerous contacts within the past 10 days, eliminating the need for a ring and thus excluding regions of the country that do not pose any risk. Another interesting approach to assessing optimal vaccination, although not specific to FMD, was taken by Keeling and Shattock (13). They investigated optimal prophylactic vaccine distribution in spatially segregated populations that minimizes the total number of expected cases by calculating the final epidemic size before vaccination using a probabilistic function. Their results showed that for a limited number of doses, the optimal policy would be to vaccinate one population at the expense of excluding others. However, as the authors discussed, this vaccine distribution would likely not be implemented even if it minimized the final epidemic size, given the ethical implications of inequitable distribution.

Other approaches have incorporated the interplay of multiple stakeholders’ actions to assess optimal vaccination (14, 15). Game theory can help stakeholders analyze their decisions, knowing that others’ decisions will impact their outcomes and vice versa (16, 17). In veterinary medicine, most producers decide to prevent, diagnose, and control infectious diseases without recognizing the effect of surrounding producers’ decisions. However, the nature of a transboundary infectious disease means that an individual producer’s prevention or control decisions may need to consider the collective decisions of others involved in their production and supply chains, even when the affected stakeholder may view responding to an infectious disease as an individual task. Silveira and Burnquist (14) applied game theory to study farmers’ strategic decisions to protect their herds in response to the risk of FMD infection in Brazil. They found that regardless of government incentives, farmers would prefer to vaccinate if vaccinated and non-vaccinated cattle did not have a differentiated market. Conversely, if non-vaccinated cattle were sold in a differentiated market with a premium of 33% or more over the base market price, the game equilibrium would shift toward non-vaccination. The study by Railey and Marsh (15) explained the low FMD vaccine uptake in East Africa by comparing their FMD vaccination game payoffs to those of a traditional vaccination game for seasonal influenza and commercial livestock vaccination. They demonstrated that vaccination provided lower payoffs than not vaccinating, supporting the current low uptake behavior and the demand for higher quality vaccines.

Epidemiological modeling and game theory are effective techniques for generating estimates of the optimal allocation of vaccines and contributing to decision-making. In the U. S., the decision to vaccinate for FMD depends on federal, state, and tribal policies. Since the U. S. is categorized as an FMD-free country and the vaccine stockpile is much smaller than the population at risk, vaccination allocations must be evaluated alongside various strategies for national and international business continuity and economic recovery. Therefore, it is essential to assess the optimal allocation of limited vaccine stockpiles across political boundaries where states and the federal government have diverse policies, logistical constraints, population demographics, and disease dynamics. We coupled epidemiological modeling with game theory to develop a stochastic framework that strategically examines decision positions on the allocation of limited FMD vaccine doses from a series of simultaneous multi-player decision sets, using desired criteria and rules of allocation. In the study, analyses of the payoffs associated with allocating vaccine doses in four states in the Midwest based on multiple stakeholders’ choices were evaluated according to equilibrium principles of game theory.

## 2 Materials and methods

### 2.1 Study population

This study used a synthetic farm dataset of the United States livestock population, provided by the United States Department of Agriculture Center for Epidemiology and Animal Health (USDA-CEAH). Briefly, the synthetic livestock population was developed using a spatial microsimulation model, the Farm Location and Agricultural Production Simulator (FLAPS) (18). FLAPS accounts for livestock census data, including production types and herd sizes, and allocates geographical coordinates consistent with county-level USDA: National Agricultural Statistics Service (NASS) livestock population census data. Locations from market and processor operations were derived from the Packers and Stockyards Act data and other publicly accessible data (19, 20). NASS collects and publicly reports farm census data by production type at the county level within the U. S. For privacy reasons, specific geolocations are not reported, necessitating a modeling process to provide geolocation for NASS census data. Additionally, for counties with fewer than three farms of a specific production type, the number of farms is not reported at the county level but aggregated for reporting across multiple counties. Therefore, FLAPS was used to simulate the distribution and populations of individual livestock farms throughout the conterminous U. S., including imputing missing farms in counties not reported (18). The model uses geographic and demographic information to disaggregate the county-level input data and create a synthetic population of individually geolocated farms by production type and county location (Supplementary Figures S1, S2). A full description of the FLAPS simulator is available (18). The provided simulated national livestock farm population data consisted of 1,176,177 operations, including 1,166,639 farm operations, 5,010 dealer operations, 2,647 market operations, and 1,881 processor operations. Farm animal numbers included 160,388 bison, 89,077,274 cattle, 87,991,215 pigs, 2,627,243 goats, and 5,342,281 sheep. Descriptive statistics of livestock farms and operation types at the national level can be seen in Supplementary Table S1.

Although bison, pigs, goats, deer, and sheep are also susceptible species to FMD, we focused the allocation of vaccine doses on cattle populations. We restricted vaccine dose allocation to four states: Iowa (IA), Minnesota (MN), Nebraska (NE), and Wisconsin (WI). IA was selected as the index state, while MN, NE, and WI were chosen as neighboring states. The number of operations, types of production, and number of animals differ substantially across states. For the four states studied here, IA had 38,509 operations and 24,084,813 farm animals; MN had 28,818 operations and 9,779,589 farm animals; NE had 27,641 operations and 9,134,857 farm animals; and WI had 32,261 operations and 2,921,388 farm animals. Descriptive statistics of livestock farms and operation types at the state level can be found in Supplementary Tables S2–S5.

### 2.2 Epidemiological model and parameters

**Figure 1:**
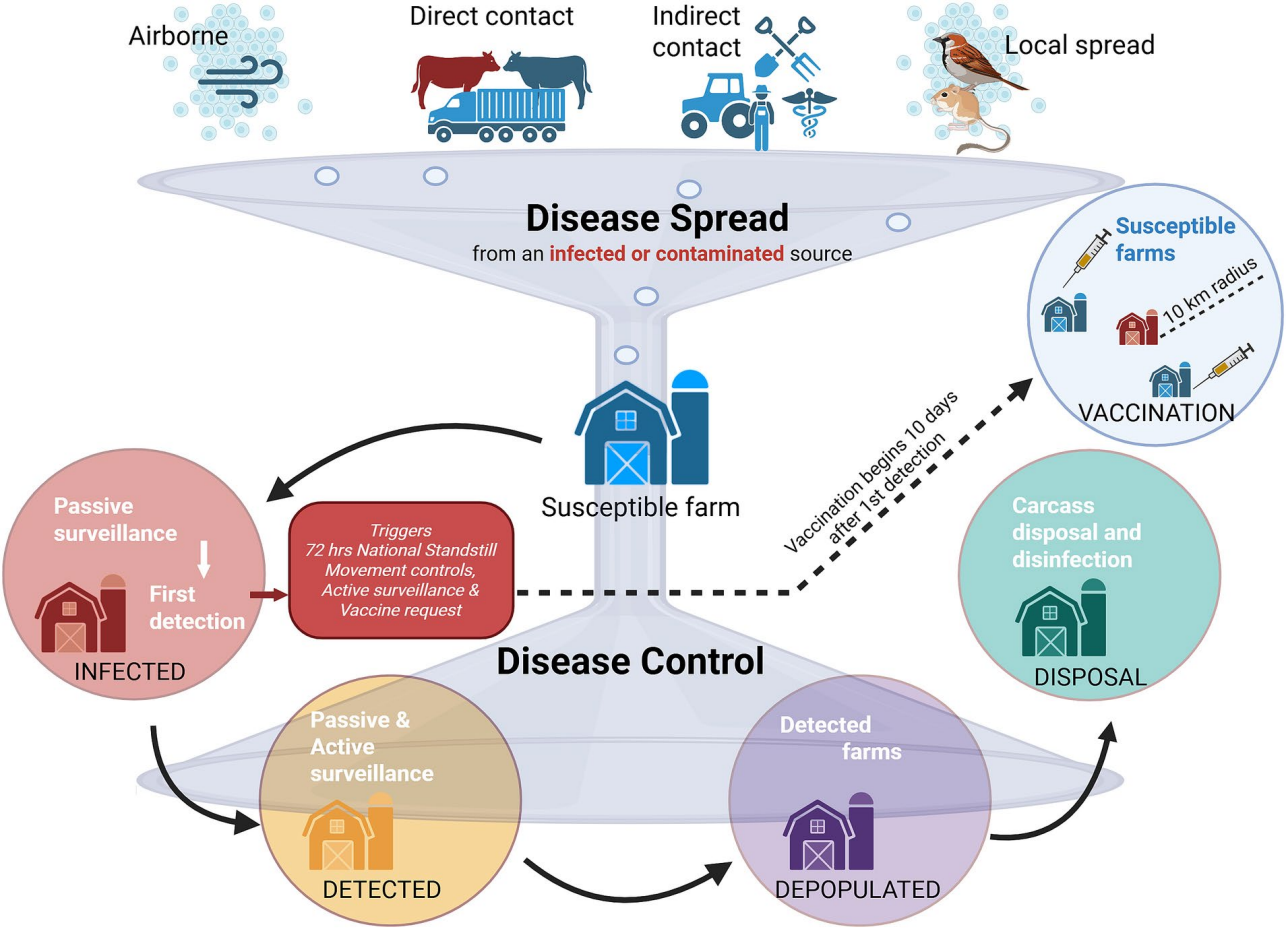
Diagram of foot-and-mouth disease spread and integrated control management simulated in the InterSpread Plus model. There are four disease spread mechanisms by which a susceptible farm can be infected. For example, the movement of infected animals (direct contact), infected farms produce daily shipments at the production type-specific Poisson rate, which are assigned to destination production types based on a destination type probability distribution and shipment distance probability distribution. Passive surveillance (based on clinical signs) results in first detection, which then triggers a 72-h national standstill, movement controls in the control area, active surveillance, and vaccine requests. Passive and active surveillance activities continue to detect farms. A susceptible farm can progress through four distinct states (infection, detection, depopulation, and disposal), but it can also transition from susceptible to vaccinated to vaccine immune. Created in BioRender. https://BioRender.com/mcbilw0.

The FMD scenarios developed were simulated using the InterSpread Plus (ISP) modeling system (Version 6.01.44) (21). ISP is a spatial and stochastic simulation model where spread mechanisms and control strategies function at the operational level (i.e., farm, market, dealer, processor), and the time-step unit is 1 day. ISP requires geolocated population data, including defined operation types (Supplementary material: workbook “ISP parameters,” tab “OperationTypes”). Direct and indirect contacts between operation types are explicitly simulated based on contact rates generated by a Poisson process, as well as the probability distributions of destination operation types and shipment distances. For example, infected farms produce daily shipments at the operation-type-specific Poisson rate, which are assigned to destination operation types based on a probability distribution for destination types and a probability distribution for shipment distances. Shipments generate new infections based on farm-type-specific infectivity and according to the number of days since the source farm was infected. Aerosol and local transmission are determined by the distance from an infected source farm and the time since the source farm showed clinical signs. Farms progress from susceptible to infected based on modeled contact (direct, indirect, local, or aerosol) with an infected premises. Once infected, farms progress to subclinical and clinical states over time based on parameters derived from published literature (22–24). Control mechanisms include movement controls to decrease the rate of direct and indirect contacts, surveillance of farms within defined surveillance zones, depopulation of infected herds with a daily capacity, and vaccination with a daily capacity (animals per day) and the duration in days of the vaccination program. Through this process, ISP simulates the FMD outbreak in the U. S., providing daily herd infections and simulating the effects of control scenarios, including the allocation of vaccination to specific herds (Figure 1).

The FMD simulated scenarios were developed using the simulated national livestock population of the United States (Supplementary Table S1). Movement characteristics of operations (i.e., farms, markets, dealers, and processors), including destinations, frequencies, and distances, are based on National Animal Health Monitoring System (NAHMS) survey data, published literature, and expert opinion, and vary based on animal type, herd size, and regional location. The U. S. continental states were categorized into five discrete regions: Pacific, Midwest, Great Lakes, Northeast, and Southeast, to account for heterogeneities in parameters (3). The spread mechanisms simulated were local spread, airborne spread, and direct and indirect contact. The herd-level infectivity parameters, specifically the incubation period, were based on previously estimated FMDV pan-serotype values for bison, cattle, and swine (22, 23) and FMDV serotype O values for sheep and goats (24). The control mechanisms exercised in all scenarios included movement controls, depopulation, surveillance, and traceability of animal movements and indirect contacts. All scenarios were allowed to spread nationally in all susceptible species. Each iteration was set to terminate when there were no new detections for 90 days. A summary of disease spread and control model parameters is provided in Supplementary material: workbook “ISP parameters.”

We simulated the introduction of FMDV on day one of the simulation in two index dairy farms located in Bremer and Benton counties in IA, with a constant silent spread phase of 14 days. Once the first farm was detected, a 72-h national movement standstill and tracing controls were implemented. Control and surveillance zones were created for each newly detected farm. In addition, a stamping-out control was initiated 24 h after the first detection. Only detected farms were depopulated, and the depopulation rate depended on animal type and herd size (Supplementary material: workbook “ISP parameters,” tab “Depopulation”). A 10 km vaccination ring around a detected premise was established when vaccination control was simulated. Only cattle operations (i.e., dairy, cow-calf, stocker, and feedlot) were selected for vaccination. The probability that an operation within the vaccination ring would be vaccinated was assumed to be 0.85. Vaccine effectiveness was assumed to be 75%, and the time for the vaccine to induce full immunity was modeled as 7 days, with partial immunity as early as 3–4 days following vaccination (Supplementary material: workbook “ISP parameters,” tab “Vaccination”).

### 2.3 Decision-making analyses

**Figure 2:**
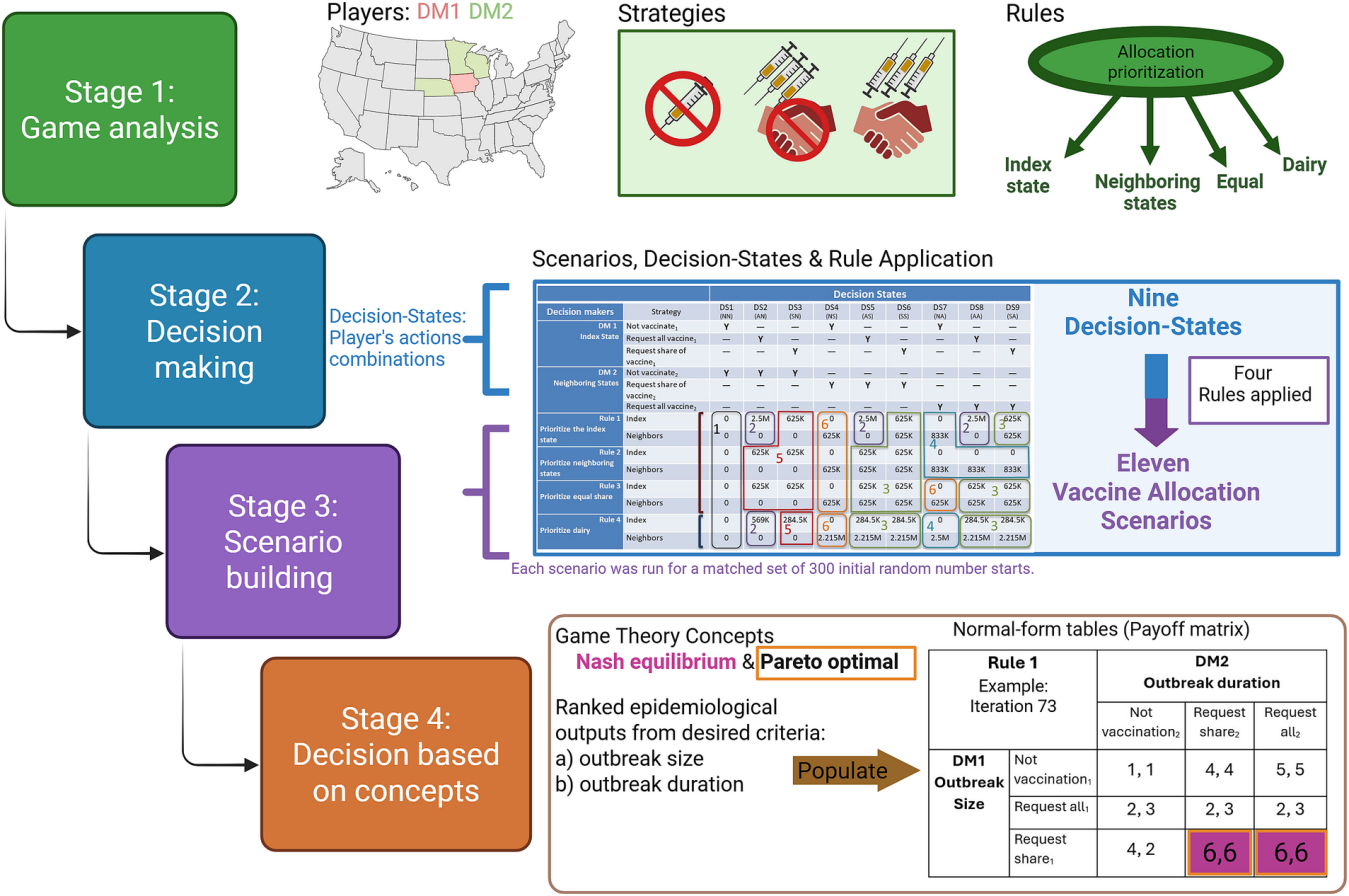
Stochastic framework incorporating epidemiological data and analyses of the payoffs of multiple stakeholders’ choices. Each stage of the analysis outlines the game’s main components and actions. Decision-makers are abbreviated as DM1 and DM2. Stage 4 is a significantly more extensive process; therefore, an extended example is provided in Supplementary material: Workbook “Stochastic game process.” Created in BioRender. https://BioRender.com/azzeb0i.

To assess the optimal allocation of vaccine doses, we developed a stochastic framework incorporating epidemiological data and analyses of the payoffs of multiple stakeholders’ choices (Figure 2). Based on game theory analyses, four main components are essential: the players, strategies, evaluation criteria, and payoffs. For this study, we identified two players or decision-makers (DMs). The index state (IA) is DM1, and the neighboring states (NE, MN, and WI) are DM2. The set of strategies for each DM contained three elements: not vaccinate (N), request a share of vaccine (S), and request all vaccine (A) (Table 1). The criteria for evaluation and the payoffs were based on the ranks of the stochastic outputs of outbreak size and outbreak duration from the matched simulated scenarios.

**Table 1:** Decision-makers and their strategy sets.

Decision-makers	Strategy^^^	Descriptions
DM 1 Index State	Not vaccinate_1_ (N)	The primary response to infectious disease outbreaks in livestock is based on movement restriction and stamping out.
	Request all vaccine_1_ (A)*	All vaccine doses available are requested and implemented in addition to the primary response.
	Request share of vaccine_1_ (S)	Vaccine doses available are shared and implemented in addition to the primary response.
DM 2 Neighboring States	Not vaccinate_2_ (N)	The primary response to infectious disease outbreaks in livestock is based on movement restriction and stamping out.
	Request all vaccine_2_ (A)*	All vaccine doses available are requested and implemented in addition to the primary response.
	Request share of vaccine_2_ (S)	Vaccine doses available are shared and implemented in addition to the primary response.

^The subscript number 1 or 2 corresponds to the strategy of decision-maker 1 (DM1) or DM2, respectively. *The difference between request all vaccine _1_ and _2_ is the number of doses allocated per state since DM1 comprises one state and DM2 three states. For example, if the index state requests all vaccine doses, it receives 2.5 million doses. However, if the neighboring states request all vaccine doses, then each of the three states receives 833,000 vaccine doses.

We assumed that a third party (USDA) would evaluate the requested vaccine doses from the DMs. Therefore, we established four rules to guide the vaccine dose allocation strategies within the model.

- Rule 1 (Index priority) prioritized vaccine dose allocation to the index state—if they request all available vaccine, they receive all available doses. If the index state requests only a share, other states receive the remaining doses. If the index state elects not to vaccinate, other states receive their full amount of requested doses.
- Rule 2 (Neighbor priority) prioritized vaccine dose allocation to the neighboring states—if they requested all doses, they received them all. If the neighboring states request only a share, the index state receives some doses, and if they elect not to vaccinate, the index state gets their requested doses. These allocations assume that the third party wants to reserve a portion of doses for potential later allocation/use.
- Rule 3 (Equal priority) prioritized sharing among all—an equal share was retained for all.
- Rule 4 (Dairy priority) prioritized vaccine dose allocation based on the dairy cattle population in each state. Under this rule, if DM1 requests all doses while DM2 does not vaccinate, then DM1 receives a number of doses corresponding to its proportion of the dairy cattle in the four states—284,500 (11.38% of the 2.5 million total doses)—plus an additional 284,500 doses. Thus, DM1 receives a total of 569,000 doses under the assumption that USDA would not allocate all 2.5 million doses to a single state with less than 12% of the dairy cattle in the four-state region (see Supplementary Tables S2–S5 for dairy cattle numbers). If DM2 requests all doses while DM1 does not vaccinate, then DM2 is given the number of doses corresponding to its proportion of the dairy cattle in the four states—88.62% of the 2.5 million total doses—plus an additional 284,500 doses. Thus, DM2 receives all 2.5 million doses. In each decision-state mentioned above, both DM1 and DM2 received an additional estimated 284,500 doses. This number corresponds to IA’s proportion of dairy cattle, 11.38%, and is selected to achieve dose increases for both decision-makers while not exceeding available doses and adhering to the stated assumption. Alternatively, we could have allocated all 2.5 million doses to each DM requesting all doses; however, prior knowledge from state-based scenarios indicated that significant vaccine waste would occur in Iowa because it cannot administer that many doses.

### 2.4 Simulated vaccine dose allocation scenarios

Two steps were taken to determine the vaccine dose allocation scenarios. First, each DM simultaneously selected one of their three strategies [not vaccinate (N), request all (A), request share (S)]. As a result, the combination of possible strategies between DMs created nine decision-states [DS1 (NN), DS2 (AN), DS3 (SN), DS4 (NS), DS5 (AS), DS6 (SS), DS7 (NA), DS8 (AA), and DS9 (SA)] (Table 2). For example, in one decision-state, DM1 chooses to request all (A) and DM2 chooses not to vaccinate (N) [DS2 (AN)] (Table 2). Second, each of the four vaccine allocation rules is applied to each decision-state (Table 3). In the previous example, where decision-state two [DS2 (AN)] is analyzed, the application of rule 1—prioritization of the index state—allocates the 2.5 million doses to DM1 and zero doses to DM2 (Table 3).

**Table 2:** Feasible decision-states.

Decision-makers	Strategy^^^	Decision-states								
		DS1 (NN)	DS2 (AN)	DS3 (SN)	DS4 (NS)	DS5 (AS)	DS6 (SS)	DS7 (NA)	DS8 (AA)	DS9 (SA)
DM 1 Index State	Not vaccinate_1_	Y	–	–	Y	–	–	Y	–	–
	Request all vaccine_1_	–	Y	–	–	Y	–	–	Y	–
	Request share of vaccine_1_	–	–	Y	–	–	Y	–	–	Y
DM 2 Neighboring States	Not vaccinate_2_	Y	Y	Y	–	–	–	–	–	–
	Request share of vaccine_2_	–	–	–	Y	Y	Y	–	–	–
	Request all vaccine_2_	–	–	–	–	–	–	Y	Y	Y

^The subscript number 1 or 2 corresponds to the strategy of decision-maker 1 (DM1) or DM2, respectively. The three strategies are abbreviated as N (for not vaccinate), A (for request all vaccine), and S (for request share of vaccine). A decision-maker may select a strategy “Y”; for example, DM1 chooses not to vaccinate (N) in DS1, DS4, and DS7. Hence the players’ action combinations determine the number of decision-states (DS). Herein, nine feasible decision-states were determined [DS1 (NN), DS2 (AN), DS3 (SN), DS4 (NS), DS5 (AS), DS6 (SS), DS7 (NA), DS8 (AA), and DS9 (SA)]. For example, DS1 (NN) means that both DMs chose not to vaccinate (NN), while for DS2 (AN), DM1 requested all vaccine (A), and DM2 chose not to vaccinate (N).

**Table 3:** Scenario building: application of the four rules of allocation to each of the nine feasible decision-states.

Decision-makers	Strategy^^^	Decision-states								
		DS1 (NN)	DS2 (AN)	DS3 (SN)	DS4 (NS)	DS5 (AS)	DS6 (SS)	DS7 (NA)	DS8 (AA)	DS9 (SA)
DM 1 Index State	Not vaccinate_1_	Y	–	–	Y	–	–	Y	–	–
	Request all vaccine_1_	–	Y	–	–	Y	–	–	Y	–
	Request share of vaccine_1_	–	–	Y	–	–	Y	–	–	Y
DM 2 Neighboring States	Not vaccinate_2_	Y	Y	Y	–	–	–	–	–	—
	Request share of vaccine_2_	–	–	–	Y	Y	Y	–	–	–
	Request all vaccine_2_	–	–	–	–	–	–	Y	Y	Y
Rule 1 state-Allocation	Index	0	2.5 M	625 K	0	2.5 M	625 K	0	2.5 M	625 K
	Neighbors	0	0	0	625 K	0	625 K	833 K	0	625 K
Rule 2 state-Allocation	Index	0	625 K	625 K	0	625 K	625 K	0	0	0
	Neighbors	0	0	0	625 K	625 K	625 K	833 K	833 K	833 K
Rule 3 state-Allocation	Index	0	625 K	625 K	0	625 K	625 K	0	625 K	625 K
	Neighbors	0	0	0	625 K	625 K	625 K	625 K	625 K	625 K
Rule 4 dairy-Allocation	Index	0	569,000	284,500	0	284,500	284,500	0	284,500	284,500
	Neighbors	0	0	0	2,215,500	2,215,500	2,215,500	2,500,000	2,215,500	2,215,500

^The subscript number 1 or 2 corresponds to the strategy of decision-maker 1 (DM1) or DM2, respectively. The three strategies are abbreviated as N (for not vaccinate), A (for request all vaccine), and S (for request share of vaccine). Rule 1—prioritizing index state; Rule 2—prioritizing neighboring states; Rule 3—equal prioritization among states; Rule 4—allocation based on state dairy cattle percentages. The “M” means millions, and the “K” means thousands. For rule 4, the total number of vaccine doses allocated was 2,500,000. The dose allocation percentage for Iowa is 22.76% for DS2 (AN) and 11.38% for DS3 (SN), DS5 (AS), DS6 (SS), DS8 (AA) and DS9 (SA). Wisconsin, Minnesota, and Nebraska, respectively, is as follows: DS4 (NS), DS5 (AS), DS6 (SS), DS8 (AA), and DS9 (SA) (62.45, 22.87, 3.3%); and DS7 (NA) (66.24, 26.66, 7.1%).

Implementing each of the four rules to each of the nine decision-states generated 11 unique dose allocation scenarios, including a baseline scenario (base-1) where stamping out and movement controls were implemented with no vaccination (Tables 4, 5). Five unique scenarios resulted from vaccination allocation divided among states according to rules 1–3. We refer to those as state-based dose allocation scenarios (Table 4). Similarly, the other five scenarios simulated the allocation of vaccine doses based on the percentage of dairy cattle in the four-state region that were in each state. We refer to these five scenarios as dairy-based dose allocation scenarios (Table 5).

**Table 4:** Simulated foot-and-mouth disease outbreaks, state-based allocation scenarios.

Scenario allocation No.	Strategy^^^	Scenario name	Decision-makers*	Initial capacity^$^	Number of targeted allocated doses	Days implemented
base-1	Not vaccinate_1,2_	baseline^@^	DM1 and DM2	–	–	–
state-2	Request all vaccine_1_	state-based selfish index	DM1	50,000	IA 2,500,000	IA 88
state-3	Request share of vaccine_1,2_	state-based cooperative neighbors and index	DM1 and DM2	50,000 and 50,000	IA 625,000^&^ WI 625,000^&^ MN 625,000^&^ NE 625,000^&^	IA 35 WI 32 MN 88 NE 65
state-4	Request all vaccine_2_	state-based selfish neighbors	DM2	50,000	WI 833,000^&^ MN 833,000^&^ NE 833,000^&^	WI 40 MN 88 NE 88
state-5	Request share of vaccine_1_	state-based cooperative index	DM1	50,000	IA 625,000^#^	IA 35
state-6	Request share of vaccine_2_	state-based cooperative neighbors	DM2	50,000	WI 625,000^#^ MN 625,000^#^ NE 625,000^#^	WI 30 MN 88 NE 65

^@^The baseline scenario is the same for the state- and dairy-based allocation scenarios. *The index state (DM1) and the neighboring states (DM2). The neighboring states’ decision-makers are composed of three states. ^$^The initial capacity (number of vaccine doses per day) implemented is by state [Iowa (IA), Wisconsin (WI), Minnesota (MN), Nebraska (NE)]. ^&^The total number of vaccine doses (2.5 M) was divided by four in scenario state-3 and by three in scenario state-4. ^#^The total number of vaccine doses (2.5 M) was divided between four states cooperatively, even when DM1 or DM2 decided not to vaccinate. ^^^Subscript 1 refers to DM1, and subscript 2 refers to DM2.

**Table 5:** Simulated foot-and-mouth disease outbreaks, dairy-based allocation scenarios.

Scenario allocation No.	Strategy^^^	Scenario name	Decision-makers*	Initial capacity^$^	Number of targeted allocated doses^&^	Days implemented
base-1	Not vaccinate_1,2_	baseline^@^	DM1 and DM2	–	–	–
dairy-2	Request all vaccine_1_	dairy-based selfish index	DM1	IA (50 K)	IA 569,000	IA 32
dairy-3	Request share of vaccine_1,2_	dairy-based cooperative neighbors and index	DM1 and DM2	IA (50 K) WI (50 K) MN (50 K) NE (10 K)	IA 284,500 WI 1,561,250 MN 571,750 NE 82,500	IA 10 WI 88 MN 88 NE 8
dairy-4	Request all vaccine_2_	dairy-based selfish neighbors	DM2	WI (50 K) MN (50 K) NE (10 K)	WI 1,656,083 MN 666,583 NE 177,333	WI 88 MN 88 NE 18
dairy-5^#^	Request share of vaccine_1_	dairy-based cooperative index	DM1	IA (50 K)	IA 284,500	IA 10
dairy-6^#^	Request share of vaccine_2_	dairy-based cooperative neighbors	DM2	WI (50 K) MN (50 K) NE (10 K)	WI 1,561,250 MN 571,750 NE 82,500	WI 88 MN 88 NE 8

^@^The baseline scenario is the same for the state- and dairy-based allocation scenarios. *The index state (DM1) and the neighboring states (DM2). The neighboring states’ decision-makers are composed of three states. ^$^The initial capacity (number of vaccine doses per day) implemented is by State [Iowa (IA), Wisconsin (WI), Minnesota (MN), Nebraska (NE)]. ^&^The number of doses allocated per State was based on the number of dairy cattle in each participating State divided by the total dairy cattle among the four participating States based on NASS Ag Census date 2020. ^#^Doses were allocated cooperatively to the requesting decision-maker, even when DM1 or DM2 decided not to vaccinate. ^^^Subscript 1 refers to DM1, and subscript 2 refers to DM2.

#### 2.4.1 State-based dose allocation scenarios

For these five scenarios, we assumed the vaccine banks would have 2.5 million doses available to deploy 10 days after the first detection and that each state would have an initial vaccination capacity of 50,000 doses per day. In addition to the vaccination assumptions mentioned in the epidemiological model and parameters section, only non-infected cattle were vaccinated, all types of cattle (dairy, cow-calf, stocker, and feedlot) were given equal vaccination prioritization, and only one dose per animal was administered.

The number of vaccine doses available is not directly parameterized in ISP. The ISP model requires three pieces of information to allocate the requested vaccine doses: (1) the type of animal to be vaccinated, (2) vaccination capacity per day, and (3) the number of days that this capacity will be maintained. For example, in scenario state-2 (Table 4), 2.5 million doses were requested by DM1; thus, with a capacity of 50,000 doses per day, 50 days of vaccination at capacity would be needed. Within each iteration, vaccine doses are used each day up to the total number of cattle available to vaccinate (within a 10 km vaccination ring around a detected premise) or the daily maximum capacity; vaccination stops after the specified number of days, regardless of whether all available vaccines are used. This process can also lead to vaccine use in excess of the targeted allocation. When vaccination begins in ISP, it is performed at the herd level, so herds are chosen for vaccination each day as long as there is remaining capacity. For example, on day 1 of the vaccination period with a capacity of 10,000 doses per day, 5 farms are chosen. After selecting 4 farms with a total population of 9,500, capacity remains, and a 5th farm is chosen. That farm will be vaccinated, and if it has more than 500 animals, the capacity for that day will be exceeded. This could happen each day. Therefore, allocating the requested doses is not the same as using the requested doses, resulting in the potential to waste or over-allocate vaccines. To overcome the ISP model limitation for vaccine allocation and optimize the intended allocation of doses, we initially ran each scenario for 50 iterations to estimate the number of days needed to use the requested doses, identifying the 90th percentiles of doses used. Based on preliminary results for the 90th percentile of vaccine use, we adjusted the days of vaccination to most closely reach the targeted allocation of vaccine doses. Additionally, because we focused on the initial allocation of vaccines available in the vaccine banks, we limited our evaluation to 98 days, based on USDA estimates available in 2020 of the time needed to get a new batch of vaccines (FMD Red Book 2020). Based on these trial runs to optimize the usage of the intended allocation, the number of days of vaccination varied by state (Table 4). For the state-3 scenario, each state requested 625,000 doses: IA implemented them for 35 days, WI for 32 days, MN for 88 days, and NE for 65 days (Table 4). All models were run for 300 iterations.

#### 2.4.2 Dairy-based dose allocation scenarios

For these five scenarios, we assumed the vaccine banks would have 2.5 million doses available to deploy 10 days after the first detection, and that Iowa, Wisconsin, and Minnesota would each have an initial vaccine capacity of 50,000 doses per day, while Nebraska would have a capacity of 10,000 doses per day. In these scenarios, dairy cattle were prioritized for vaccination over cow-calf, stocker, and feedlot cattle. Other vaccination assumptions mentioned in the epidemiological model and parameters section remained unchanged, including that only non-infected cattle were vaccinated, and one dose per animal was administered.

While the state-based allocation scenarios were based on the number of states requesting vaccines, the dairy-based allocation scenarios were determined by the number of dairy cattle in each requesting state. There were 2,528,368 dairy cattle among the four states studied. IA accounted for 11.38%, MN for 22.87%, NE for 3.3%, and WI for 62.45% of the dairy cattle (Supplementary Tables S2–S5). We used results from the state-based cooperative neighbors and index scenario to initially guide the number of days required to allocate the targeted doses for each state (Supplementary Table S6). Based on the state-based cooperative neighbors and index scenario and preliminary results for the 90th percentile of vaccine use in the dairy-based scenarios, we adjusted the vaccination days to most closely reach the targeted allocation of vaccine doses. In these scenarios, the number of vaccination days varied by state. For example, in scenario dairy-3 (Table 5), where both DMs chose to vaccinate and share, 10 days were allocated to use approximately 284,500 doses requested in IA, 88 days for approximately 1,561,250 doses requested in WI, 88 days for approximately 571,750 doses requested in MN, and 8 days for approximately 82,500 doses requested in NE. We used the 90th percentiles to assess the overall use of doses per scenario. All models were run for 300 iterations.

### 2.5 Epidemiological outcomes, ranking, and normal-form tables

In our stochastic framework, each decision-maker must select criteria to evaluate their outbreak situation. We evaluated two criteria: outbreak duration and outbreak size. Outbreak duration was defined as the number of days from the first detection to the last depopulation for each DM in the game, and outbreak size was defined as the number of infected farms for each DM. These criteria were assessed at the DM level; for example, DM1 evaluated their criteria within IA, while DM2 evaluated their criteria by grouping outcomes from WI, MN, and NE. Analyses of the outcomes of the stochastic epidemiological scenarios were conducted in R version 4.2.0 (25) in RStudio (26).

Each iteration was initiated with a fixed random number, which was consistently applied across scenarios. ISP generates the starting random number for each iteration in a scenario in advance, so for 300 iterations, 300 random numbers are generated during initiation to be sequentially used for starting each of the 300 iterations. Using the same fixed random number across different scenarios results in the same random number sequences for initiating iterations used in all scenarios. This allowed us to compare results for the same iteration of each state- or dairy-based allocation scenario and to rank the outcomes. Ranking was performed using the rank function version 3.6.2 of the base package in R (25); ties were resolved with the “max” method. For instance, starting with iteration one for the baseline scenario and the five state-based allocation scenarios summarized in Table 4 (which all began with the same random number), we ranked the results for each scenario and each criterion for each decision-maker. Since there are six scenarios, ranks range from 1 to 6, with larger numbers indicating better outcomes (fewer infected farms, shorter outbreak duration). Thus, the highest size rank for a decision-maker was assigned to the scenario with the fewest infected farms, the highest duration rank to the shortest outbreak duration, the lowest size rank to the scenario with the greatest number of infected farms, and the lowest duration rank to the scenario with the longest outbreak duration. We repeated this process for each of the 300 iterations of the baseline and state-based allocation scenarios, as well as for the 300 iterations of the baseline and dairy-based allocation scenarios (Table 5), resulting in ranks for each DM in each iteration.

**Figure 3:**
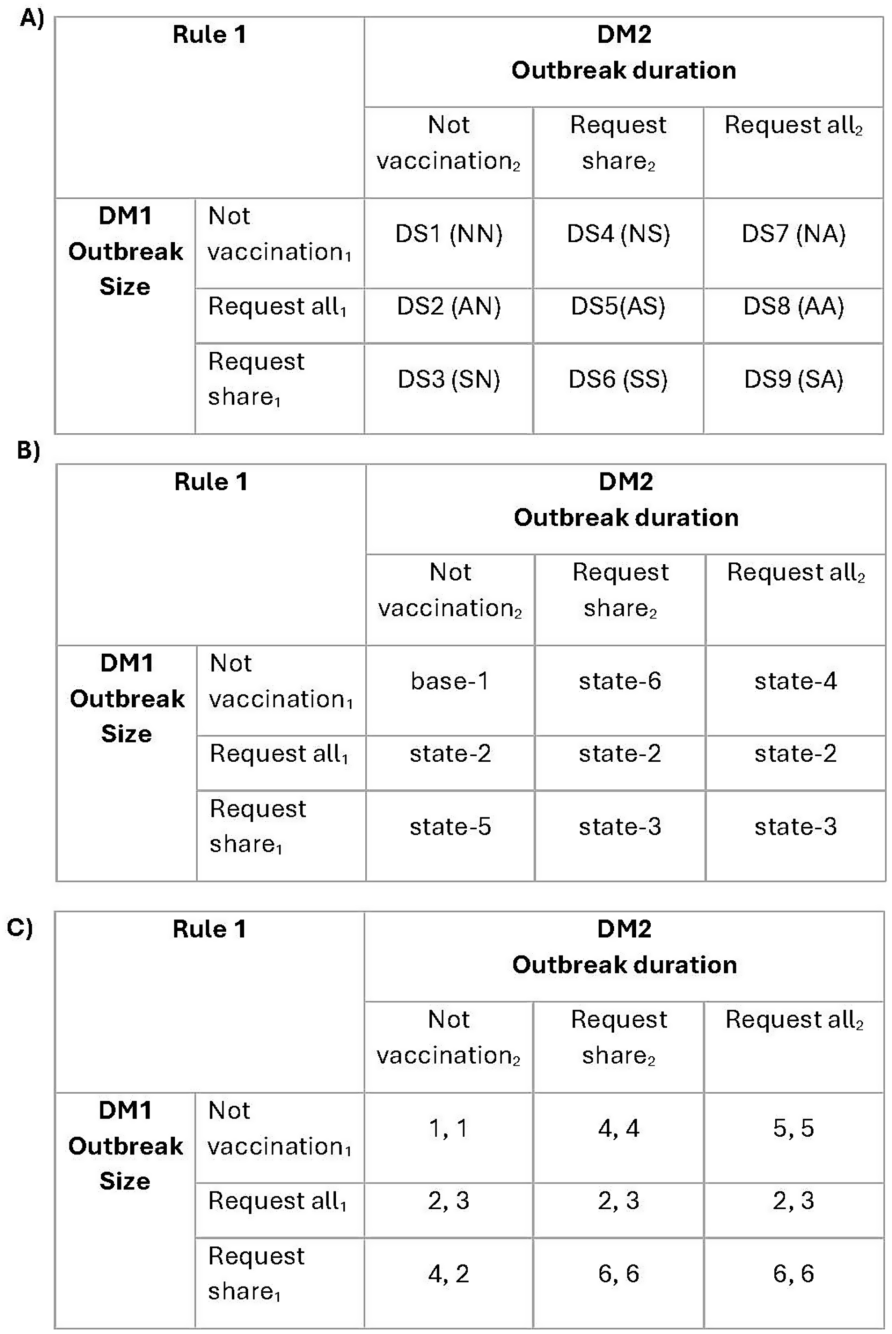
Illustration of a normal-form table. **(A)** A normal-form table showing the positioning of the nine decision-states (DS1–DS9). The rows represent the strategies for DM1, and the columns represent the strategies for DM2. For example, DS1 (NN) means that DM1 and DM2 choose not to vaccinate (N), and DS5 (AS) means DM1 chooses to request all vaccines (A) while DM2 chooses to request a share of the vaccines (S). **(B)** A normal-form table showing the mapping of the simulated scenarios of each of the nine decision-states for Rule 1 (index priority) (also see Table 14). **(C)** A normal-form table showing the payoffs of each of the nine decision-states. In this example, the payoffs of the ranked iteration of 73 epidemiological outcomes (outbreak size for DM1 and outbreak duration for DM2) of Rule 1 were selected. The first number in the cell is the payoff for DM1, and the second is the payoff for DM2.

In the game theory framework, the rankings provide the payoffs associated with decision-states and were used to populate normal-form tables (see an example in Supplementary material: workbook “Stochastic game process,” tab “ExampleStage4Process”). A normal-form table is a matrix representation of a game in which the rows represent DM1’s strategies and the columns represent DM2’s strategies, with each cell (decision-state [DS]) containing the payoffs of DM1 and DM2, respectively (Figure 3). Each of the nine decision-states in a game corresponds to one of the simulated scenarios (Figure 3). A distinct normal-form table was created for each iteration, each decision rule, and each selected criteria combination. Hence, a total of 3,600 normal-form tables were assembled [(300 iterations) × (4 allocation rules) × (3 criteria combinations)]. In any normal-form table, the payoffs to DM1 or DM2 are determined by the various selected criteria combinations of outbreak size and duration rankings. The selected criteria combinations demonstrate the framework’s flexibility rather than an exhaustive evaluation of all possible criteria or their combinations, as decision-makers would select criteria based on their goals during an outbreak. The 3,600 normal-form tables were created using R version 4.2.0 (25) in RStudio (26).

### 2.6 Analyzing Nash equilibrium and Pareto optimal solutions

Each normal-form table was analyzed for Nash equilibria and Pareto optimal solutions. We used the sim-nasheq function of the Recon package (27) for Nash equilibrium and the psel function of the rPref package (28) for Pareto optimality in R. An outcome is a Nash equilibrium if neither decision-maker can change their own decision to increase their own payoff, regardless of the impact on the other. An outcome is Pareto optimal if there is no other outcome where both decision-makers are better off.

In brief, each normal-form table represented a game under an allocation rule and consisted of nine decision-states (players’ action combinations) along with the payoffs from one iteration for each decision-state for the studied rule. Analyses identified which of the nine decision-states constituted a Nash equilibrium and/or a Pareto optimal solution under those payoffs for each normal-form table. This process was repeated for all 300 normal-form games associated with that vaccine allocation rule and each of the different criteria combinations. This same sequence was completed for all four decision rules. Thus, incorporating the stochastic epidemiological outcomes into the game analyses allowed us to calculate the fraction of games in which a particular decision-state (combination of choices by the DMs) was a Nash equilibrium and/or a Pareto optimal solution for each allocation rule and criteria combination. The nine decision-states resolved to six unique vaccine allocation scenarios (states 1–6) across rules 1–3 and six unique vaccine allocation scenarios (dairy 1–6) for rule 4.

## 3 Results

### 3.1 Descriptive statistics of the foot-and-mouth disease simulated scenarios

#### 3.1.1 Descriptive statistics at the national level

The foot-and-mouth disease outbreaks simulated in this project report the spread from two large dairy farms (dairy1 *n* = 2901; dairy2 *n* = 851) in IA, the index state, to neighboring and non-neighboring U. S. states. The median number of infected farms at first detection (14 days) was 22 at the national level, with 21 of those within IA, MN, NE, and WI (Supplementary Table S7). The baseline scenario’s national outbreak size and duration, where movement and stamping out controls were implemented, had a median of 802 infected farms and a median duration of 198 days (Supplementary Tables S8, S9). The majority were cattle farms, with a median of 720 infected farms. All cattle production types were infected in most iterations; large feedlots were the least frequently infected (Supplementary Table S10). The median number of infected animals was 790,158 (Supplementary Table S11), with most of these being cattle (median of 683,312) and swine (median of 59,237). At the national level, most vaccination scenarios had a modest effect, with a numerically lower median number of infected farms and shorter outbreak duration compared to the baseline scenario (Supplementary Tables S8, S9). Additionally, vaccination scenarios consistently had fewer infected farms at the 75th percentile compared to the baseline scenario (Supplementary Table S8). These results represent the effect of the vaccines allocated to the four states in the game. We did not allocate vaccines to any states that were not participants in the game.

#### 3.1.2 Descriptive statistics of the baseline scenario for the index state (DM1) and neighboring states (DM2)

Descriptive statistics for outbreak size and duration for state-based allocation scenarios are presented in Tables 6, 7, respectively, and in Supplementary Tables S12, S13. Descriptive statistics for outbreak size and duration for dairy-based allocation scenarios are presented in Tables 8, 9, respectively, and in Supplementary Tables S14, S15. In the baseline scenario (base-1), the index state, IA, had a median of 141 infected farms, while the neighboring states (NE, WI, MN) had a median of 292 infected farms (Tables 6, 8). Individual neighboring states had the following medians for infected farms: NE had 54, WI had 149, and MN had 73 (Supplementary Tables S12–S14). Iowa had a median outbreak duration of 140 days, and the neighboring states had a median of 166 days (Tables 7, 9). Individual neighboring states had the following medians for outbreak duration: NE 144 days, WI 135 days, and MN 127 days (Supplementary Tables S13–S15). For visualization of the tabular data, see Supplementary Figures S3–S14.

**Table 6:** Descriptive statistics of outbreak size (number of infected farms) of the state-based allocation scenarios for the index state (DM1) and neighboring states (DM2).

No.	Scenario	DM1	25%	Median	Mean	75%	90%
base-1	baseline	IA	72	141	140	190	240
state-2	state-based selfish index	IA	68	110	115	155	194
state-3	state-based cooperative neighbors and index	IA	63	105	112	152	207
state-4	state-based selfish neighbors	IA	74	122	128	183	224
state-5	state-based cooperative index	IA	67	116	120	159	207
state-6	state-based cooperative neighbors	IA	75	126	132	182	232
		DM2					
base-1	baseline	NE_WI_MN	112	292	294	447	551
state-2	state-based selfish index	NE_WI_MN	98	258	273	408	562
state-3	state-based cooperative neighbors and index	NE_WI_MN	86	190	217	323	453
state-4	state-based selfish neighbors	NE_WI_MN	98	218	236	349	469
state-5	state-based cooperative index	NE_WI_MN	106	254	278	430	554
state-6	state-based cooperative neighbors	NE_WI_MN	100	240	251	381	483

Unique vaccine allocation scenarios (state-based 1–6) for rules 1–3.

**Table 7:** Descriptive statistics of outbreak duration of the state-based allocation scenarios for the index state (DM1) and neighboring states (DM2).

No.	Scenario	DM1	25%	Median	Mean	75%	90%
base-1	baseline	IA	90	140	156	212	259
state-2	state-based selfish index	IA	80	126	137	187	225
state-3	state-based cooperative neighbors and index	IA	74	113	134	186	235
state-4	state-based selfish neighbors	IA	81	131	139	191	222
state-5	state-based cooperative index	IA	81	126	142	195	232
state-6	state-based cooperative neighbors	IA	86	131	144	195	235
		DM2					
base-1	baseline	NE_WI_MN	109	166	177	229	278
state-2	state-based selfish index	NE_WI_MN	99	148	158	216	254
state-3	state-based cooperative neighbors and index	NE_WI_MN	90	132	152	210	256
state-4	state-based selfish neighbors	NE_WI_MN	99	144	155	212	245
state-5	state-based cooperative index	NE_WI_MN	102	149	161	215	255
state-6	state-based cooperative neighbors	NE_WI_MN	100	152	158	212	249

Unique vaccine allocation scenarios (state-based 1–6) for rules 1–3.

**Table 8:** Descriptive statistics of outbreak size (number of infected farms) of the dairy-based allocation scenarios for the index state (DM1) and neighboring states (DM2).

No.	Scenario	DM1	25%	Median	Mean	75%	90%
base-1	baseline	IA	72	141	140	190	240
dairy-2	dairy-based selfish index	IA	70	116	122	165	213
dairy-3	dairy-based cooperative neighbors and index	IA	72	118	124	172	216
dairy-4	dairy-based selfish neighbors	IA	70	123	126	175	220
dairy-5	dairy-based cooperative index	IA	71	120	128	182	230
dairy-6	dairy-based cooperative neighbors	IA	70	124	127	172	222
		DM2					
base-1	baseline	NE_WI_MN	112	292	294	447	551
dairy-2	dairy-based selfish index	NE_WI_MN	113	250	282	419	567
dairy-3	dairy-based cooperative neighbors and index	NE_WI_MN	98	210	234	341	466
dairy-4	dairy-based selfish neighbors	NE_WI_MN	103	223	230	338	427
dairy-5	dairy-based cooperative index	NE_WI_MN	113	266	285	428	563
dairy-6	dairy-based cooperative neighbors	NE_WI_MN	103	222	233	348	440

Unique vaccine allocation scenarios (dairy-based 1–6) for rules 4.

**Table 9:** Descriptive statistics of outbreak duration of the dairy-based allocation scenarios for the index state (DM1) and neighboring states (DM2).

No.	Scenario	DM1	25%	Median	Mean	75%	90%
base-1	baseline	IA	90	140	156	212	259
dairy-2	dairy-based selfish index	IA	85	135	144	195	234
dairy-3	dairy-based cooperative neighbors and index	IA	78	127	139	192	231
dairy-4	dairy-based selfish neighbors	IA	81	120	136	193	227
dairy-5	dairy-based cooperative index	IA	89	140	153	209	251
dairy-6	dairy-based cooperative neighbors	IA	81	122	137	197	235
		DM2					
base-1	baseline	NE_WI_MN	109	166	177	229	278
dairy-2	dairy-based selfish index	NE_WI_MN	101	157	164	220	254
dairy-3	dairy-based cooperative neighbors and index	NE_WI_MN	93	149	156	217	253
dairy-4	dairy-based selfish neighbors	NE_WI_MN	99	139	153	209	250
dairy-5	dairy-based cooperative index	NE_WI_MN	104	168	170	226	268
dairy-6	dairy-based cooperative neighbors	NE_WI_MN	99	140	154	211	258

Unique vaccine allocation scenarios (dairy-based 1–6) for rules 4.

In the baseline scenario (base-1), FMDV spread from IA to MN in 98% (295/300) of the simulations, to NE in 93% (278/300) of the simulations, and to WI in 100% of the simulations (300/300). Specific farm type infection risks are reported in Supplementary Table S16.

#### 3.1.3 Descriptive statistics of the state-based vaccine allocation scenarios for the index state (DM1) and neighboring states (DM2)

**Figure 4:**
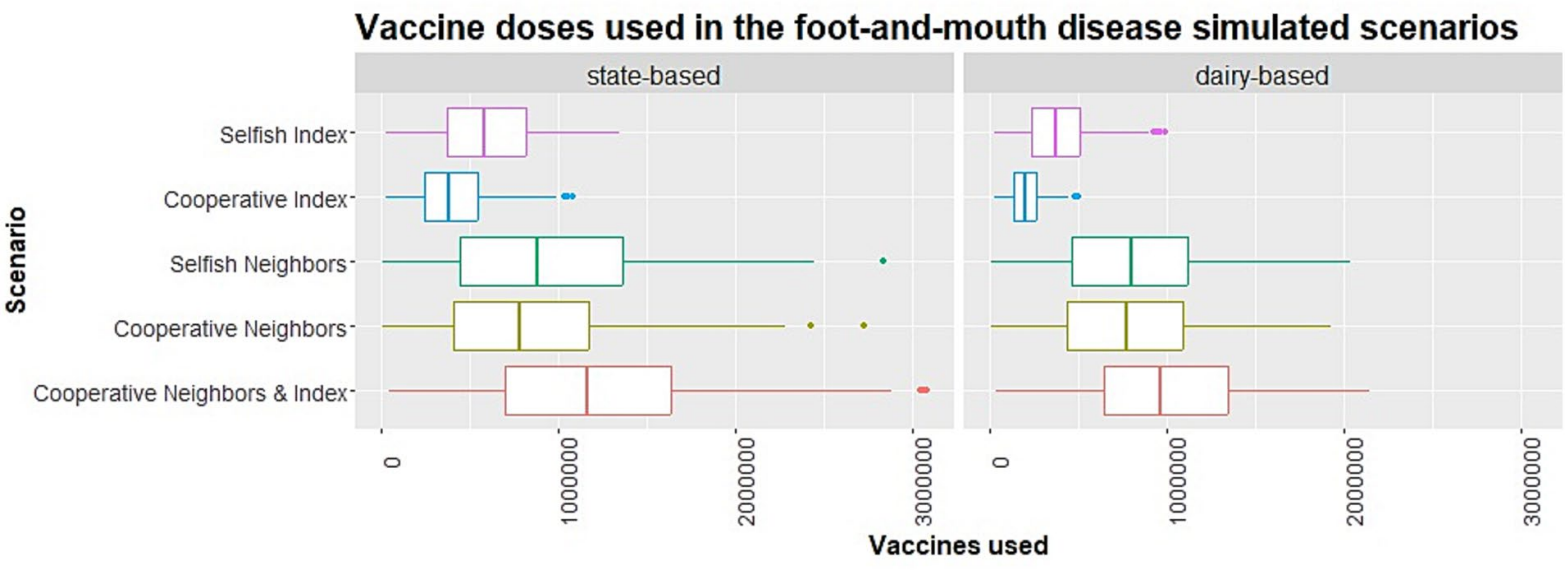
Boxplots of vaccine doses used for all states in the state- and dairy-based allocation scenarios.

Five state-based vaccination allocation scenarios were simulated based on rules 1–3 and 2.5 million available vaccine doses. The vaccine allocation scenarios resulted in small to modest decreases in the number of infected herds and duration of infection compared to the baseline no vaccination scenario (base-1). The descriptive summary of the state-based vaccine allocation scenarios’ outbreak metrics demonstrated some differences between scenarios but did not clearly identify a scenario that consistently achieved the smallest outbreak size and duration across quantiles (Tables 6–7, for visualization of the tabular data, see Supplementary Figures S3, S5, S7, S8, S11, S12). The state-based selfish index scenario (state-2) simulated the allocation of all 2.5 million doses to the index state, resulting in a median of 578,740 doses used and a 90th percentile of 1,016,409 doses used, representing 41% of the 2.5 million available doses (Tables 10, 11). For most other allocations, vaccine usage at the 90th percentile for IA, NE, MN, and WI nearly matched the allocated doses (Tables 10, 11). For the selfish neighbors scenario (state-4), the 90th percentile of MN vaccine doses used was substantially less than the doses allocated. Details on the vaccine doses used per scenario can be seen in Figure 4 and in Supplementary Table S17.

**Table 10:** Descriptive statistics of vaccine doses used on the state-based allocation scenarios.

No.	Scenario	State	25%	Median	Mean	75%	90%
state-2	state-based selfish index	IA	372,170	578,740	598,344	818,812	1,016,409
state-3	state-based cooperative neighbors and index	IA	255,061	402,070	413,631	538,712	695,730
state-3	state-based cooperative neighbors and index	MN	90,178	188,244	226,552	331,956	491,258
state-3	state-based cooperative neighbors and index	NE	105,796	233,197	302,221	433,214	654,879
state-3	state-based cooperative neighbors and index	WI	145,312	302,808	316,628	439,242	597,799
state-4	state-based selfish neighbors	MN	97,151	229,457	250,108	363,914	489,008
state-4	state-based selfish neighbors	NE	107,300	298,656	380,932	574,979	877,818
state-4	state-based selfish neighbors	WI	169,368	359,077	375,036	539,747	676,974
state-5	state-based cooperative index	IA	242,268	381,722	408,343	548,803	686,159
state-6	state-based cooperative neighbors	MN	99,250	236,312	253,693	371,240	512,864
state-6	state-based cooperative neighbors	NE	96,270	255,300	327,571	492,585	717,784
state-6	state-based cooperative neighbors	WI	137,135	275,793	301,883	436,474	561,727

**Table 11:** Assessing vaccine doses of the state-based allocation scenarios.

No.	Scenario	State	Capacity per day	Days	Targeted doses	Doses used 90th percentile	Percent used 90th percentile
state-2	state-based selfish index	IA	50 K	88	2,500,000	1,016,409	41
state-3	state-based cooperative neighbors and index	IA	50 K	35	625,000	695,730	111
state-3	state-based cooperative neighbors and index	MN	50 K	88	625,000	491,258	79
state-3	state-based cooperative neighbors and index	NE	50 K	65	625,000	654,879	105
state-3	state-based cooperative neighbors and index	WI	50 K	32	625,000	597,799	96
state-4	state-based selfish neighbors	MN	50 K	88	833,000	489,008	59
state-4	state-based selfish neighbors	NE	50 K	88	833,000	877,818	105
state-4	state-based selfish neighbors	WI	50 K	40	833,000	676,974	81
state-5	state-based cooperative index	IA	50 K	35	625,000	686,159	110
state-6	state-based cooperative neighbors	MN	50 K	88	625,000	512,864	82
state-6	state-based cooperative neighbors	NE	50 K	65	625,000	717,784	115
state-6	state-based cooperative neighbors	WI	50 K	30	625,000	561,727	90

#### 3.1.4 Descriptive statistics of the dairy-based vaccine allocation scenarios for the index state (DM1) and neighboring states (DM2)

Five vaccination allocation scenarios were simulated based on the fourth rule (dairy allocation) and 2,500,000 available doses. The dairy-based selfish index scenario (dairy-2) simulated that 22.76% of doses would be allocated to the index state, resulting in a median of 372,635 doses used and a 90th percentile of 646,665 doses, representing 114% of the 569,000 targeted doses (Tables 12, 13). The dairy vaccine allocation scenarios led to small decreases in the number of infected herds and the duration of infection compared to the baseline for the index state, and small to modest decreases in the neighboring states compared to the no vaccination scenario. A descriptive summary of the dairy-based vaccine allocation scenarios’ outbreak metrics demonstrated some differences between scenarios but did not clearly identify a scenario that consistently achieves the smallest outbreak size and duration across quantiles (Tables 8, 9, for visualization of the tabular data, see Supplementary Figures S4, S6, S9, S10, S13, S14). Except for WI allocations, vaccine use at the 90th percentile in the dairy-based allocation scenarios nearly matched the targeted allocation doses (Tables 12, 13). Details on the vaccine doses used per scenario can be seen in Figure 4 and Supplementary Table S17.

**Table 12:** Descriptive statistics of vaccine doses used on the dairy-based allocation scenarios.

No.	Scenario	State	25%	Median	Mean	75%	90%
dairy-2	dairy-based selfish index	IA	238,428	372,635	388,599	512,701	646,665
dairy-3	dairy-based cooperative neighbors and index	IA	137,321	204,276	212,878	267,594	347,530
dairy-3	dairy-based cooperative neighbors and index	MN	105,716	216,589	244,004	345,280	497,395
dairy-3	dairy-based cooperative neighbors and index	NE	10,992	27,562	36,243	63,138	78,150
dairy-3	dairy-based cooperative neighbors and index	WI	275,138	495,994	502,251	711,140	912,199
dairy-4	dairy-based selfish neighbors	MN	99,920	224,968	247,668	357,614	504,889
dairy-4	dairy-based selfish neighbors	NE	22,841	57,466	70,906	117,658	158,787
dairy-4	dairy-based selfish neighbors	WI	281,592	521,540	517,591	761,020	904,159
dairy-5	dairy-based cooperative index	IA	136,558	204,276	212,740	267,594	347,445
dairy-6	dairy-based cooperative neighbors	MN	99,920	230,290	250,549	364,194	521,442
dairy-6	dairy-based cooperative neighbors	NE	10,595	27,100	36,135	63,275	78,140
dairy-6	dairy-based cooperative neighbors	WI	281,592	535,093	518,847	763,224	901,246

**Table 13:** Assessing vaccine doses of the dairy-based allocation scenarios.

No.	Scenario	State	Capacity per day	Days	Targeted doses	Doses used 90th percentile	Percent used 90th percentile
dairy-2	dairy-based selfish index	IA	50 K	32	569,000	646,665	114
dairy-3	dairy-based cooperative neighbors and index	IA	50 K	10	284,500	347,530	122
dairy-3	dairy-based cooperative neighbors and index	MN	50 K	88	571,750	497,395	87
dairy-3	dairy-based cooperative neighbors and index	NE	10 K	8	82,500	78,150	95
dairy-3	dairy-based cooperative neighbors and index	WI	50 K	88	1,561,250	912,199	58
dairy-4	dairy-based selfish neighbors	MN	50 K	88	666,583	504,889	76
dairy-4	dairy-based selfish neighbors	NE	10 K	18	177,333	158,787	90
dairy-4	dairy-based selfish neighbors	WI	50 K	88	1,656,083	904,159	55
dairy-5	dairy-based cooperative index	IA	50 K	10	284,500	347,445	122
dairy-6	dairy-based cooperative neighbors	MN	50 K	88	571,750	521,442	91
dairy-6	dairy-based cooperative neighbors	NE	10 K	8	82,500	78,140	95
dairy-6	dairy-based cooperative neighbors	WI	50 K	88	1,561,250	901,246	58

### 3.2 Stochastic game results of Nash equilibrium and Pareto optimal solutions

The stochastic outcomes of the FMD vaccine allocation scenarios provided the payoffs to populate the nine decision-states for each of the 300 normal-form tables under each rule and criteria combination. Therefore, we had 3,600 normal-form tables [(300 iterations) × (4 allocation rules) × (3 criteria combinations)]. Each normal-form table contained rankings from one matched iteration (same initial random seed) from each vaccine allocation to decision-states within a rule and criteria combination. From this, the frequency of Nash equilibria and Pareto optimal outcomes per decision-state was determined to assess robustness in the decision-making process for each decision-state and criteria combination.

Table 14 summarizes the proportion of Nash equilibria and Pareto optimal outcomes for each decision-state and combination of decision criteria. Within a rule, the Nash equilibrium generally favored the DM that the rule prioritized. For Rule 1, which prioritized allocation to the index state, the most common Nash equilibrium solution is DS2 (AN) (59, 59, and 53%, respectively, for the criteria combination), in which DM1 requests all the vaccine and DM2 chooses not to vaccinate. The corresponding vaccine allocation for this decision-state under Rule 1 is to allocate all vaccine to the index state (state-2). Similarly, for Rule 2, which prioritized neighbor states, the most common Nash equilibrium solution is DS7 (NA) (52, 55, and 52%, respectively, for the criteria combination), in which DM1 chooses not to vaccinate and DM2 requests all the vaccine. The corresponding vaccine allocation for this decision-state under Rule 2 is to allocate all vaccine to the neighboring states (state-4). For Rule 3, where equal sharing between states is prioritized, the most common Nash equilibrium solutions are DS5 (AS), DS6 (SS), DS8 (AA), and DS9 (SA) (49, 54, and 47%, respectively, for the criteria combination). The corresponding vaccine allocation for these decision-states under Rule 3 is to share vaccine allocation among all participating states (state-3). The most common Pareto optimal decision-states differ from the most common Nash equilibria for Rules 1 and 2. For Rule 1 (Index priority), DS6 (SS) and DS9 (SA) are the most common Pareto optimal outcomes, while for Rule 2 (Neighbor priority), DS5 (AS) and DS6 (SS) are the most common. The corresponding vaccine allocation is to share vaccine allocation (state-3). For Rule 3, which prioritized sharing the vaccine, DS5 (AS), DS6 (SS), DS8 (AA), and DS9 (SA) are the Pareto optimal solutions. The corresponding vaccine allocation for these decision-states under Rule 3 is to share vaccine allocation among all participating states (state-3). For Rules 1 through 3, the Nash equilibria and Pareto optimal solutions were consistent across combinations of evaluation criteria (number of infected premises and outbreak duration). Across Rules 1–3, the most common Pareto optimal decision-states correspond to the vaccine allocation state-3, sharing the vaccine among all states. Furthermore, the differences in the 90th percentiles of outbreak size and duration between Nash and Pareto solutions were modest, with DM2 generally benefiting more from the Pareto solution, most notably for outbreak size under Rule 1. There was no difference in the 90th percentiles of outbreak size and duration between Nash and Pareto solutions for Rule 3 (Supplementary Table S18).

**Table 14:** The frequency of Nash equilibria and Pareto optimal solutions for each decision-state under the four rules and a selected combination of criteria.

Decision-state^#^	Rule*	Scenario allocation number^&^	DM1 OS^^^ and DM2 OD^^^		DM1 OS^^^ and DM2 OS^^^		DM1 OD^^^ and DM2 OD^^^	
			Nash equilibrium (%)	Pareto Optimal (%)	Nash equilibrium (%)	Pareto Optimal (%)	Nash equilibrium (%)	Pareto Optimal (%)
DS1 (NN)	1	base-1	24	30	23	27	28	27
DS2 (AN)	1	state-2	59	40	59	36	53	36
DS3 (SN)	1	state-5	32	38	29	33	33	35
DS4 (NS)	1	state-6	24	34	24	31	27	34
DS5 (AS)	1	state-2	44	40	44	36	39	36
DS6 (SS)	1	state-3	40	50	44	52	41	45
DS7 (NA)	1	state-4	26	34	25	34	28	34
DS8 (AA)	1	state-2	45	40	45	36	41	36
DS9 (SA)	1	state-3	40	50	44	52	40	45
DS1 (NN)	2	base-1	27	32	26	29	31	28
DS2 (AN)	2	state-5	29	41	24	35	30	36
DS3 (SN)	2	state-5	29	41	24	35	30	36
DS4 (NS)	2	state-6	28	34	27	32	32	35
DS5 (AS)	2	state-3	43	51	45	52	41	46
DS6 (SS)	2	state-3	43	51	45	52	41	46
DS7 (NA)	2	state-4	52	35	55	36	52	35
DS8 (AA)	2	state-4	37	35	41	36	37	35
DS9 (SA)	2	state-4	37	35	41	36	37	35
DS1 (NN)	3	base-1	29	32	29	32	33	29
DS2 (AN)	3	state-5	38	43	31	38	36	39
DS3 (SN)	3	state-5	38	43	31	38	36	39
DS4 (NS)	3	state-6	32	36	32	36	36	38
DS5 (AS)	3	state-3	49	52	54	55	47	49
DS6 (SS)	3	state-3	49	52	54	55	47	49
DS7 (NA)	3	state-6	32	36	32	36	36	38
DS8 (AA)	3	state-3	49	52	54	55	47	49
DS9 (SA)	3	state-3	49	52	54	55	47	49
DS1 (NN)	4	base-1	24	32	24	28	30	28
DS2 (AN)	4	dairy-2	32	36	30	35	31	29
DS3 (SN)	4	dairy-5	29	35	30	31	28	30
DS4 (NS)	4	dairy-6	37	39	37	38	43	39
DS5 (AS)	4	dairy-3	45	45	48	45	46	40
DS6 (SS)	4	dairy-3	46	45	49	45	46	40
DS7 (NA)	4	dairy-4	40	41	40	41	45	41
DS8 (AA)	4	dairy-3	44	45	47	45	44	40
DS9 (SA)	4	dairy-3	45	45	48	45	45	40

^^^OS, outbreak size; OD, outbreak duration; DM, decision-maker. ^#^N denotes not vaccinate, A denotes request all vaccine, and S denotes request share of vaccine. ^&^Specific information for the scenario allocation is displayed in Tables 4, 5. *Rule 1 (Index priority), Rule 2 (Neighbor priority), Rule 3 (Equal priority), and Rule 4 (Dairy priority). Shaded cells in green highlight the decision-states with high frequency of Nash Equilibrium and/or Pareto Optimal solutions.

For Rule 4—which prioritizes vaccine allocation based on the percentage of dairy cattle in each state—the most common Nash equilibria are DS5 (AS) and DS6 (SS), which correspond to a shared vaccine allocation among all states. The most common Pareto optimal outcomes varied across evaluation criteria, but generally, allocations that distributed vaccines among all states according to their dairy percentage [DS5 (AS), DS6 (SS), DS8 (AA), and DS9 (SA)] had the highest percentage of Pareto optimal solutions. Under Rule 4 (Dairy priority), the highest proportion of solutions that were equilibria or Pareto optimal was just under 50%, with a generally small range from the lowest to highest. Furthermore, when both DMs vaccinated according to Nash equilibrium or Pareto optimal solutions, the 90th percentiles of outbreak size and duration were the same for the first two criteria combinations (first criteria combination: DM1 outbreak size, DM2 outbreak duration; second criteria combination: DM1 and DM2 outbreak duration,) and a modest overall decrease was observed for the third criteria combination (DM1 and DM2 outbreak duration) (Supplementary Table S18).

## 4 Discussion

One of the challenges when responding to an FMD outbreak in disease-free regions is the availability of various resources associated with depopulation, movement control, and vaccination. In the case of vaccines, an initial finite supply would be available, and their potential to mitigate the size or severity of an outbreak would, in part, be determined by the optimization of the allocation of those initial doses. However, the optimal allocation of vaccine doses has been underexplored, and to our knowledge, there is no framework for strategizing their allocation across states in the U. S. (29). This study developed a stochastic framework that strategically examines decision positions on the allocation of vaccine doses from a series of simultaneous multi-player decision sets using epidemiological modeling and game theory.

Decision-makers (DMs) may have different objectives when determining their control strategies (30). In our scenarios, DMs’ objectives were related to the outcomes of the outbreak in their respective states and were thus inherently distinct. Additionally, we selected two related but distinct criteria: outbreak size and duration. In the stochastic game theory framework reported here, we analyzed each criterion for each DM. We transformed the criteria into ranks to allow comparison despite each outcome criterion having different units (days vs. number of herds). We defined outbreak size as the number of farms infected, but the number of animals infected or other criteria could also be used. Criteria serve as decision-makers’ proxies for evaluating their control actions toward achieving their goals. We chose the number of infected farms because the initial outbreak investigation occurs at the farm level, and the response is applied to the whole farm. While farm size may influence resource allocation at the intra-state level, it is beyond the scope of this framework, which focuses on inter-state resource allocation. Variability in farm size can be observed across the four states in Supplementary Tables S2–S5. Median herd size is similar across the four states, so we do not expect our results to change. For instance, the number of infected animals at the national and four-state levels is lowest in the state-based cooperative neighbors and index scenario (state-3) (see Supplementary Tables S11, S19, respectively), consistent with our farm-based analysis.

In our vaccination scenarios, we chose to vaccinate only cattle, including any farm class. The index farms were dairy cattle, and results from the baseline scenario corroborated that the spread was primarily among cattle. The FMD Red Book (4) recommends prioritizing cattle for vaccination among the different susceptible species due to their low viral threshold of infection and susceptibility. Previous research has suggested that in some countries, vaccinating only cattle could be as effective as vaccinating all species (9). However, this study did not compare the outcomes of vaccine allocation among other species. Nevertheless, knowledge gained from the UK 2001 FMD outbreak highlighted the epidemiological roles that different species can play in the spread; for example, sheep acted as silent spreaders, pigs as amplifiers, and cattle as indicators (31). Thus, analyses of scenarios considering vaccine allocation to other susceptible species could provide valuable insights for preparedness.

One difficulty in allocating the requested vaccines using ISP (Version 6.01.44) was the lack of a direct parametrization of the total number of doses available. Therefore, we estimated vaccine use over time based on 50 initial iterations and vaccine usage at the 90th percentile. In our attempt to closely approximate the targeted vaccine doses by using 50,000 or 10,000 vaccines per day, we chose to vary the duration of the vaccination program to achieve nearly 100% of the target allocation. Thus, in some cases, the potential for over-allocating vaccines is evident in Tables 11, 13. Another limitation was the number of user-defined regions (e.g., U. S. states) that could be parameterized with their respective sets of vaccine resources and strategies. We distinctly parameterized four locations (U. S. states) for vaccination (IA, NE, WI, and MN). Expanding options for parameterizing more nuanced vaccination strategies or application rates could result in different optimal strategies. However, balancing flexibility and rapid decision-making during a response can be challenging, and we believe that the scenarios explored here represent reasonable approximations of real vaccination allocation decisions.

The four allocation rules were used to demonstrate possible ways state and federal officials might distribute vaccines in response to states’ requests. We confined our analyses to prioritizing the index state, neighboring states, equal sharing, or allocation according to dairy cattle numbers in each state. The framework is readily adaptable to other allocation rules and provides insight into whether the choice of decision rule impacts epidemiological metrics. Applying this stochastic framework for game theory analysis to other regions in the country could provide general rules for guidance in vaccine allocation based on geographic, population, resource availability, and other characteristics. Additionally, examining other initial conditions, such as animal density, index farm type, and predominant farm type in the region, along with varying demographics, could enhance the robustness of the rules. Moreover, including varying capacities (i.e., vaccinated cattle per day) representative of each state could better match resource heterogeneity at the state level. Additionally, because we focused on the initial allocation of vaccines available in the vaccine banks, we limited our evaluation to 98 days based on the time needed to obtain a new batch of vaccines. However, with the new vaccine bank and ongoing vaccine planning work, the initially available vaccine or time required to get a new batch of vaccines may change. The advantage of this framework is that it is easily adapted to different initial conditions or specific regions, allowing exploration of the various factors that could affect optimal vaccine allocation.

All epidemiological models have limitations. In ISP, disease spread and non-vaccine control parameters are assigned at the regional or national levels. Thus, it is challenging to parameterize and simulate the effects of individual states’ responses to their specific livestock populations and management practices. Currently, parameterization of player/state-based disease management parameters is not possible in ISP due to limitations in the number of user-defined states that can be specified. In addition, the framework only includes a combination of two criteria and two players (e.g., the index state and neighboring states). Although adapting this framework to include more than one criterion per player and more players may be possible, it has not yet been developed. Decision-states and allocation scenarios expand dramatically as the number of players increases.

Among other factors, early or late detection has been identified as an indicator of the magnitude of epidemics (32, 33). We set the first detection to 14 days. In the baseline simulated outbreaks, the national median number of infected farms at the end of the epidemic was 802, with a median duration of 198 days. Modeling research has indicated that vaccination can reduce the epidemiological magnitude of widespread FMD outbreaks (8, 34), especially when early vaccination is considered. Metrics such as the number of infected farms at first detection can support decision-making by indicating the potential severity of an outbreak. In our study, the median number of infected farms at first detection (14 days) was 22 nationwide, 15 for IA, 4 for WI, 2 for MN, and 1 for NE (Supplementary Table S7). While the median national epidemiological outcomes show a significant outbreak in the United States, state-level results vary notably. In the United States, outbreak response is primarily executed at the state and tribal levels; thus, each state has its own plan and resource capabilities. For example, states with fewer farms infected at first detection may have the personnel and resources necessary to respond immediately to all those farms, but states with a larger number of infected farms may not have enough personnel and resources to act promptly, and the delay in response may lead to further spread of FMDV. Therefore, knowledge of the number of farms infected at first detection may be a useful metric for guiding which states to prioritize for vaccination. In this study, we selected players/states based on their proximity to the index state, rather than the number of farms infected at first detection. Given that the index state in our scenarios had the majority of infected premises at first detection, an allocation toward the player/state with the most infected premises may be approximated by our rule 1, decision-states 2, 5, and 8, allocation state-2. However, these decision-states were not the Pareto optimal solution.

Standard analysis of the total number of infected premises and outbreak duration in days did not provide a clear indication of the optimal allocation policy. As detailed in Tables 6–9, outbreak size and duration were only modestly decreased by any of the vaccination allocation strategies. In this study, we were not analyzing specific scenarios, but rather the decision-states that account for the allocation to each decision-maker. A standard analysis often struggles to show differences between allocation strategies due to the nature of the outbreak distributions, with the main differences typically only evident at the upper percentiles. In addition, standard analysis comparing allocation strategies must choose whether to minimize the median or minimize the risk of an extreme event, such as outbreak sizes at the 90th percentile. Furthermore, standard analysis requires the selection of a single output for analysis, either the index state outcome, the neighbor state’s outcome, or the combined outcome (see Supplementary Tables S20A,B for an illustration of this limitation). It does not allow for the joint incorporation of different outcomes for each decision-maker. This is the advantage of a multi-criteria or game theory analysis—the ability to assess multiple outcomes and the decisions of multiple players to determine the best course for all, rather than just one (30).

Game theory has been used extensively to inform policymakers in public health and water resource management conflicts (16, 35–38), but is less often applied to animal health. A scoping review identified 31 English-language studies on animal health, of which 27 examined decisions about infectious diseases, 22 used mathematical models, and 30 used economic output as their payoff (17). As in our study, some of these studies can inform policy recommendations. However, none propose a framework for optimal vaccine allocation or for using epidemiological metrics as payoffs. In addition, the stochastic analysis in the framework reported here incorporates the variability of epidemiological modeling outcomes by analyzing matched iterations in multiple normal-form tables and reporting the proportion of Nash equilibria and Pareto optimal decision-states for each potential allocation. This innovative approach ranked the results of each matched iteration of outbreak size and duration from the simulated scenarios and used them as payoffs to populate the normal-form table games. Each normal-form table was analyzed as a static game with perfect information. Nash equilibria and Pareto optimal solutions were analyzed across decision-states and iterations within each rule. This approach more effectively accounts for the multiple decision-makers’ preferred outcomes and the uncertainty regarding the examined strategy.

Nash equilibrium can be interpreted to advise each DM on the best response strategy to the choices of the other DMs. It can predict how DMs would adjust their behavior based on that of other DMs in the game. It can also be seen as a self-enforcing agreement because it is in a DM’s self-interest to follow the agreement if others do (39). Results from our Nash equilibrium analyses notably favored the DM prioritized by the rule, highlighting the importance of the selected rules and their potential to create tension between DMs. When there is tension between DMs’ objectives or incentives, Nash equilibrium solutions and socially desirable outcomes may differ. The prisoners’ dilemma, a classic game theory model, illustrates this point. In that model, cooperation between decision-makers results in a better outcome for both players than the non-cooperative Nash equilibrium, yet the cooperative solution is unstable because either player can improve their payoff by deviating from that solution, making cooperation a challenging task to maintain (39).

While Nash equilibrium strategies are driven by self-interest, Pareto optimal results can be understood as the strategies that represent the best trade-offs between the players’ utilities. A Pareto optimal solution is one in which no change will simultaneously improve utility for both DMs. Our results demonstrated that, for rules 1, 2, and 3, decision-states corresponding to allocations of shared vaccine between DMs were Pareto optimal solutions. This result was most stable under Rule 3 (Equal Priority). Our findings suggest that shared vaccine is the optimal and most stable allocation for both decision-makers. For Rule 4 (Dairy Priority), Pareto optimal solutions varied depending on the combination of criteria used. Additionally, for Rule 4, there is less differentiation in both outbreak duration and outbreak size among the different decision-states. This results in similar likelihoods that multiple decision-states are Pareto optimal solutions.

## 5 Conclusion

New insights using a stochastic framework that integrates game theoretical analyses with epidemiological modeling outcomes are promising avenues for supporting decision-making on the optimal allocation of vaccine doses. In the current study, this approach was applied to the allocation of vaccines during a modeled outbreak of FMD in the United States. Across all rules, shared allocation between states was frequently a Pareto optimal outcome, where any change in decision (choice to vaccinate and/or willingness to share) does not result in improved outcomes for both decision-makers. Rule 3 (Equal priority) and shared allocation of vaccines to states resulted in the most consistent Nash equilibria and Pareto optimal outcomes. In that sense, Rule 3 (Equal priority) is more likely to yield favorable societal-level outcomes even when states are making self-interested decisions. If the USDA’s objective is to minimize both outbreak size and duration for the index and neighboring states, then the state-based allocation, state-3, with shared allocation between states generally accomplishes that, since the corresponding solutions are most likely to be Pareto optimal. Differences between the 90th deciles of the baseline and state-3 scenarios show that DM1 had 34 fewer farms infected and a 22-day reduction in duration. Similarly, DM2 had 108 fewer farms infected and a 23-day reduction in duration (see Supplementary Figures S7, S8, S11, S12). This framework can assess vaccine allocation given simultaneous multi-player vaccine requests, accounting for multiple objectives reflected in the selected criteria. This aids in overcoming the difficulty of assessing the cardinal outcomes from the epidemiological scenarios and perhaps providing solutions favorable at the state, tribal, and national levels. It provides policymakers with a tool to assess the optimal allocation of vaccines requested by multiple decision-makers and has the potential to increase the likelihood of outbreak control through successful intervention (see Supplementary material, workbook “Stochastic game process,” tab “PuttingItAllTogether”). Although the current study focused exclusively on vaccination for FMD in the U. S., this stochastic framework could readily be expanded to other transboundary infectious diseases and regions or countries.

## Data Availability

The original contributions presented in the study are included in the article/Supplementary material, further inquiries can be directed to the corresponding authors.
