# Supplementary material for: A stochastic framework to assess the optimal allocation of limited vaccine doses in foot-and-mouth disease outbreaks using game theory
Source: Front Vet Sci. 2026 Feb 9;13:1681056. doi: 10.3389/fvets.2026.1681056 (PMC12930634; doi:10.3389/fvets.2026.1681056)
Supplement: Supplementary file 3 [file Image_1.pdf]

Supplementary material FIGURES: A stochastic framework to assess the optimal allocation of limited vaccine doses in foot-and-mouth disease outbreaks using game theory

Population maps

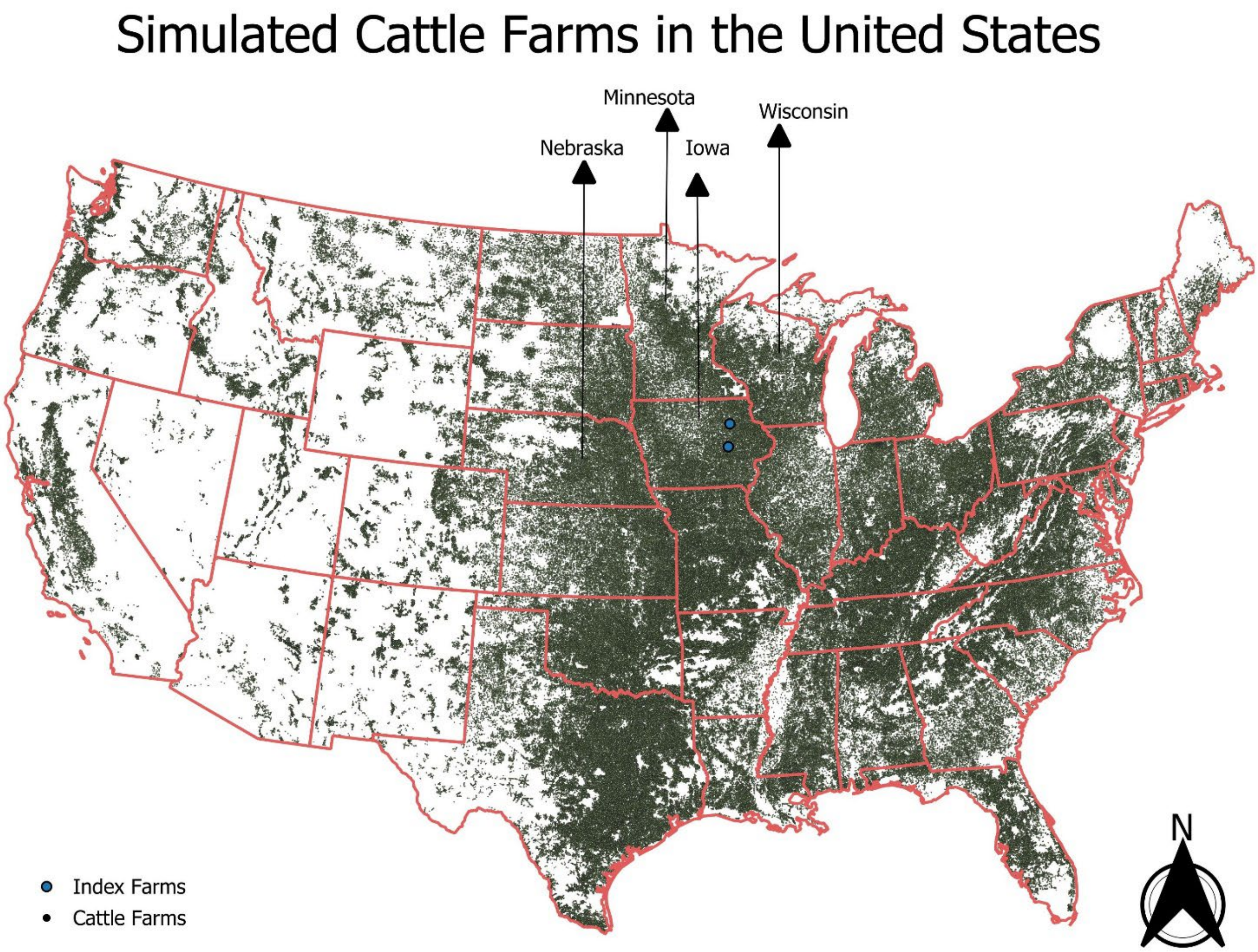

Figure S1. Distribution of the synthetic cattle farms in the conterminous United States.

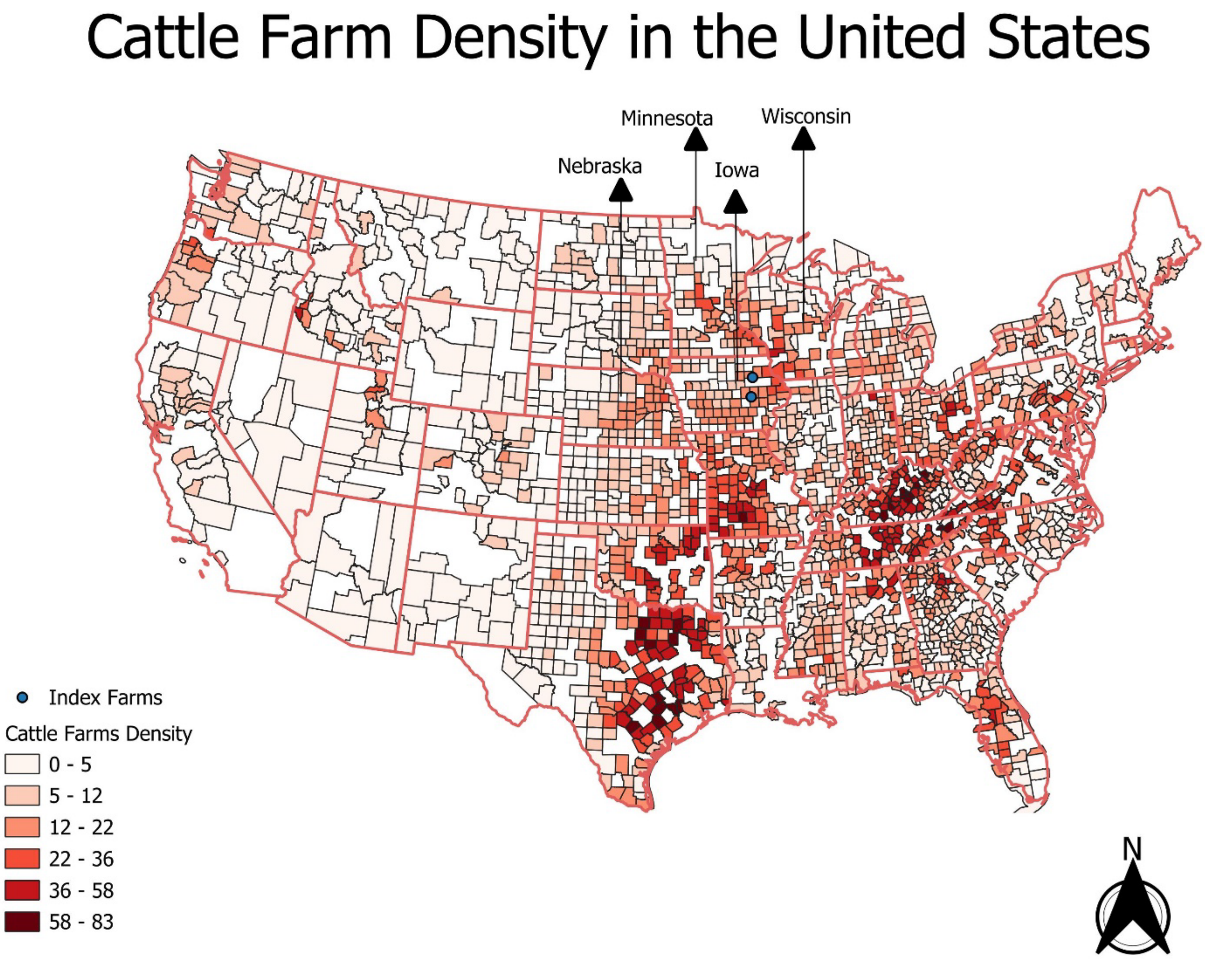

Figure S2. Cattle farm density per 30 sq miles at the county level of the synthetic cattle farms in the conterminous United States.

The following plots are derived from data in Tables 6-9 of the manuscript.

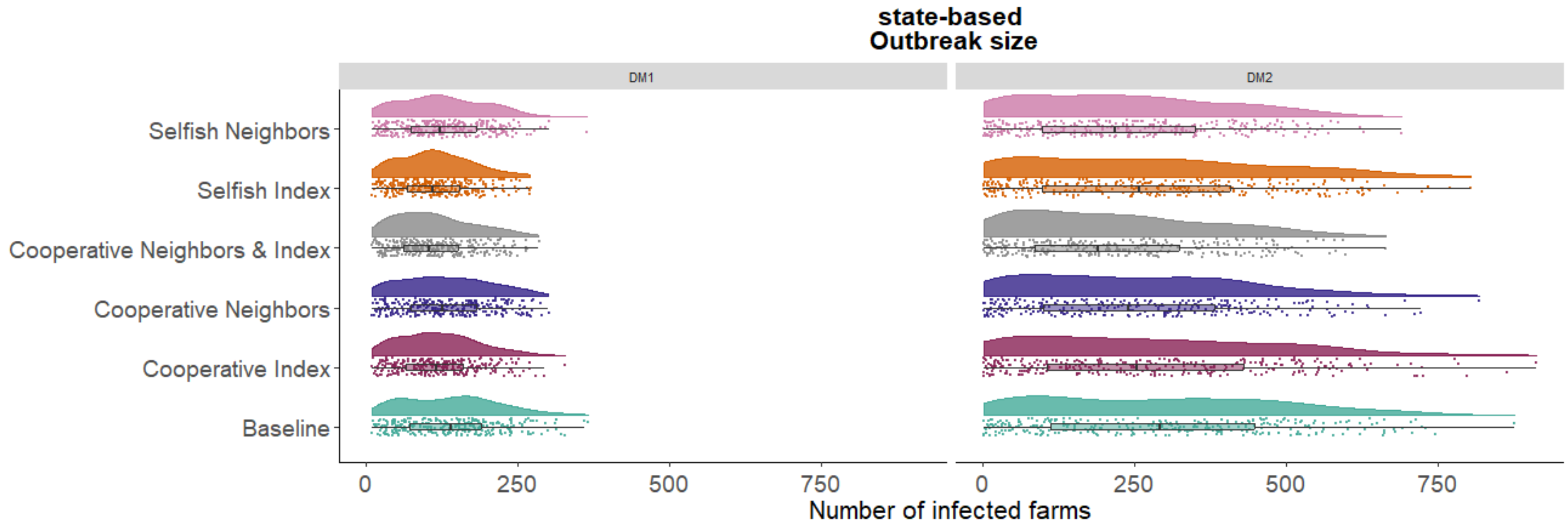

Figure S3. Number of infected farms for the state-based scenarios, by decision maker (DM1 Index state and DM2 Neighboring states).

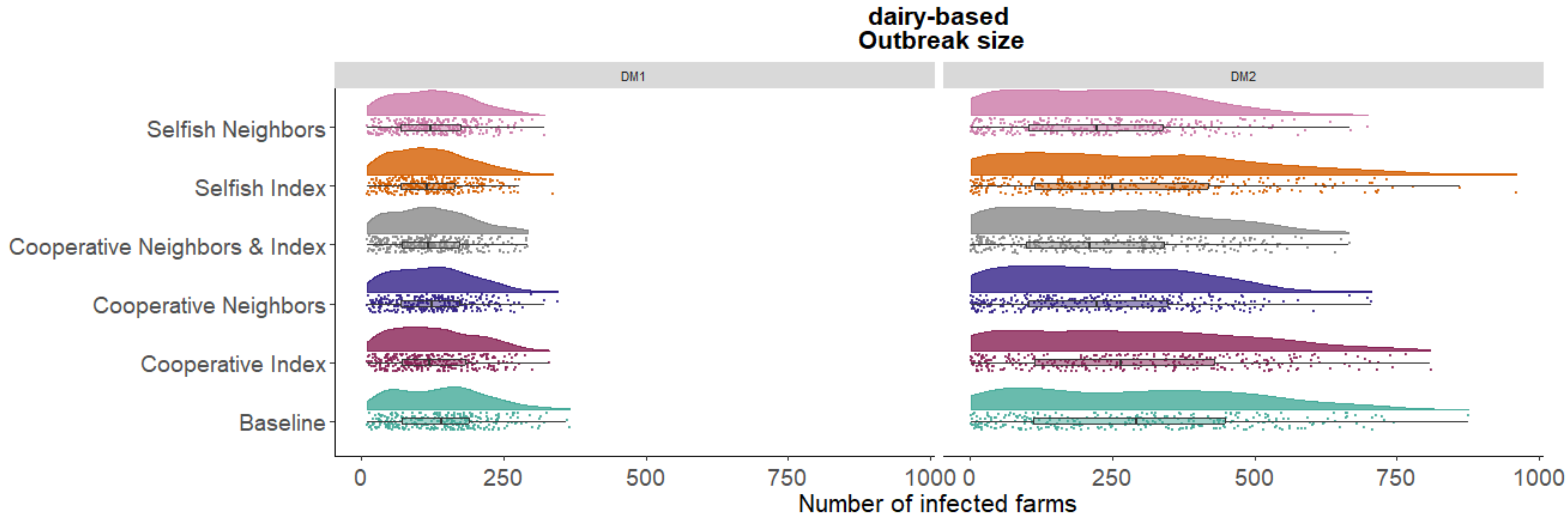

Figure S4. Number of infected farms for the dairy-based scenarios, by decision maker (DM1 Index state and DM2 Neighboring states).

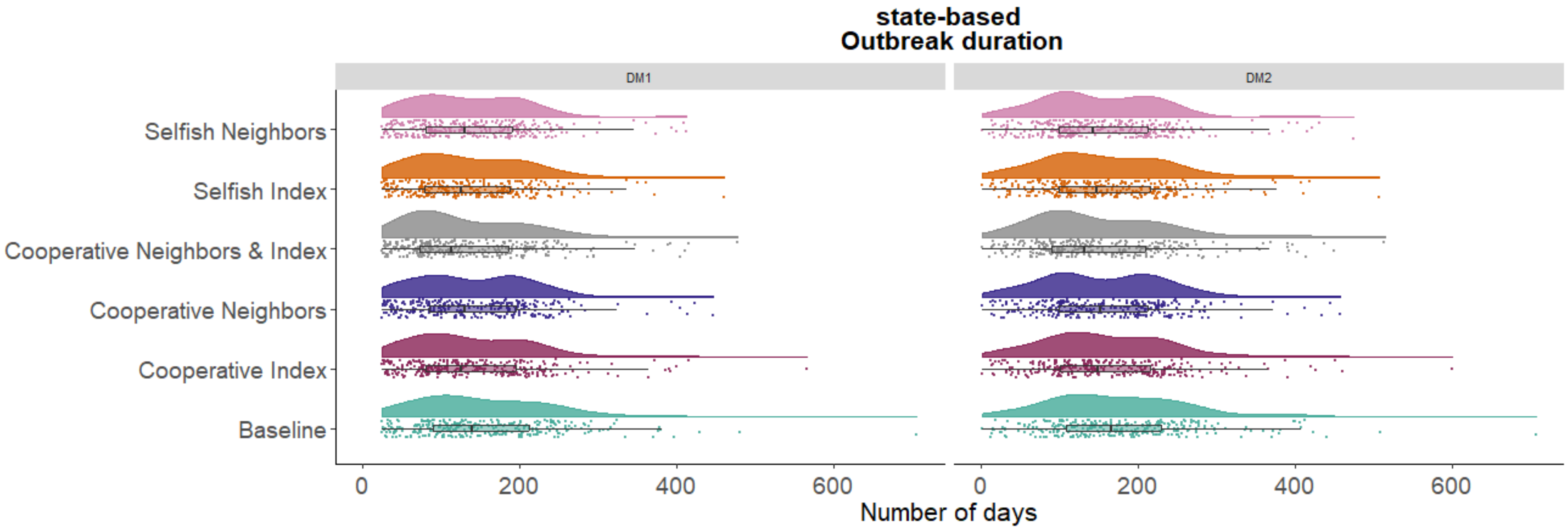

Figure S5. Outbreak duration in days for the state-based scenarios, by decision maker (DM1 Index state and DM2 Neighboring states).

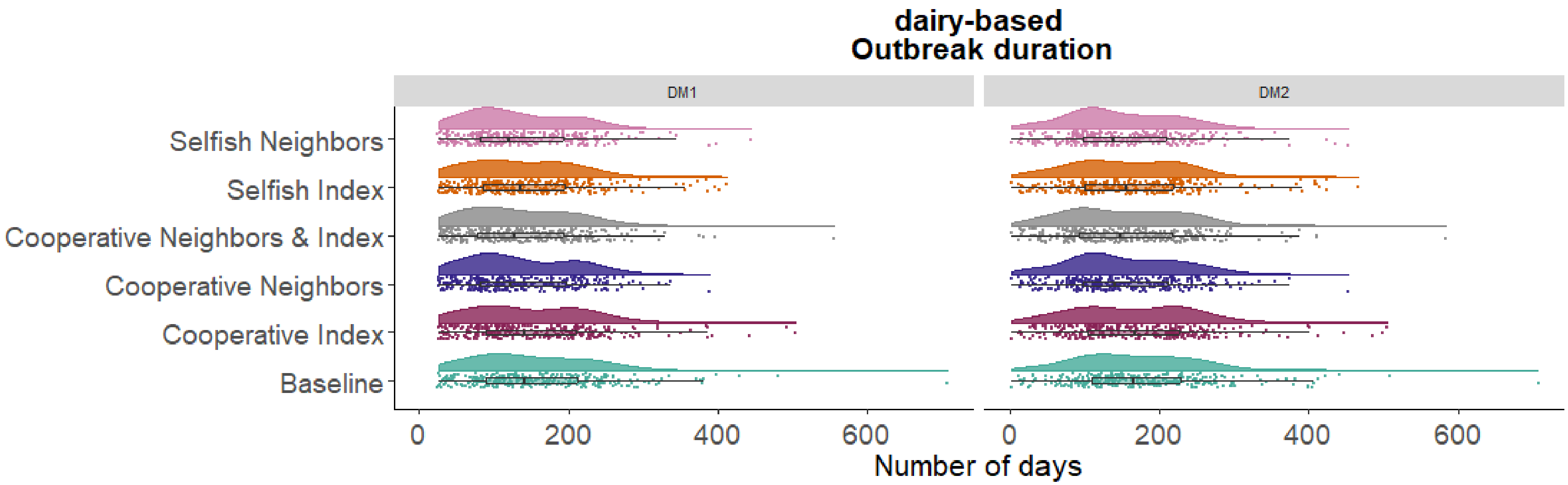

Figure S6. Outbreak duration in days for the dairy-based scenarios, by decision maker (DM1 Index state and DM2 Neighboring states).

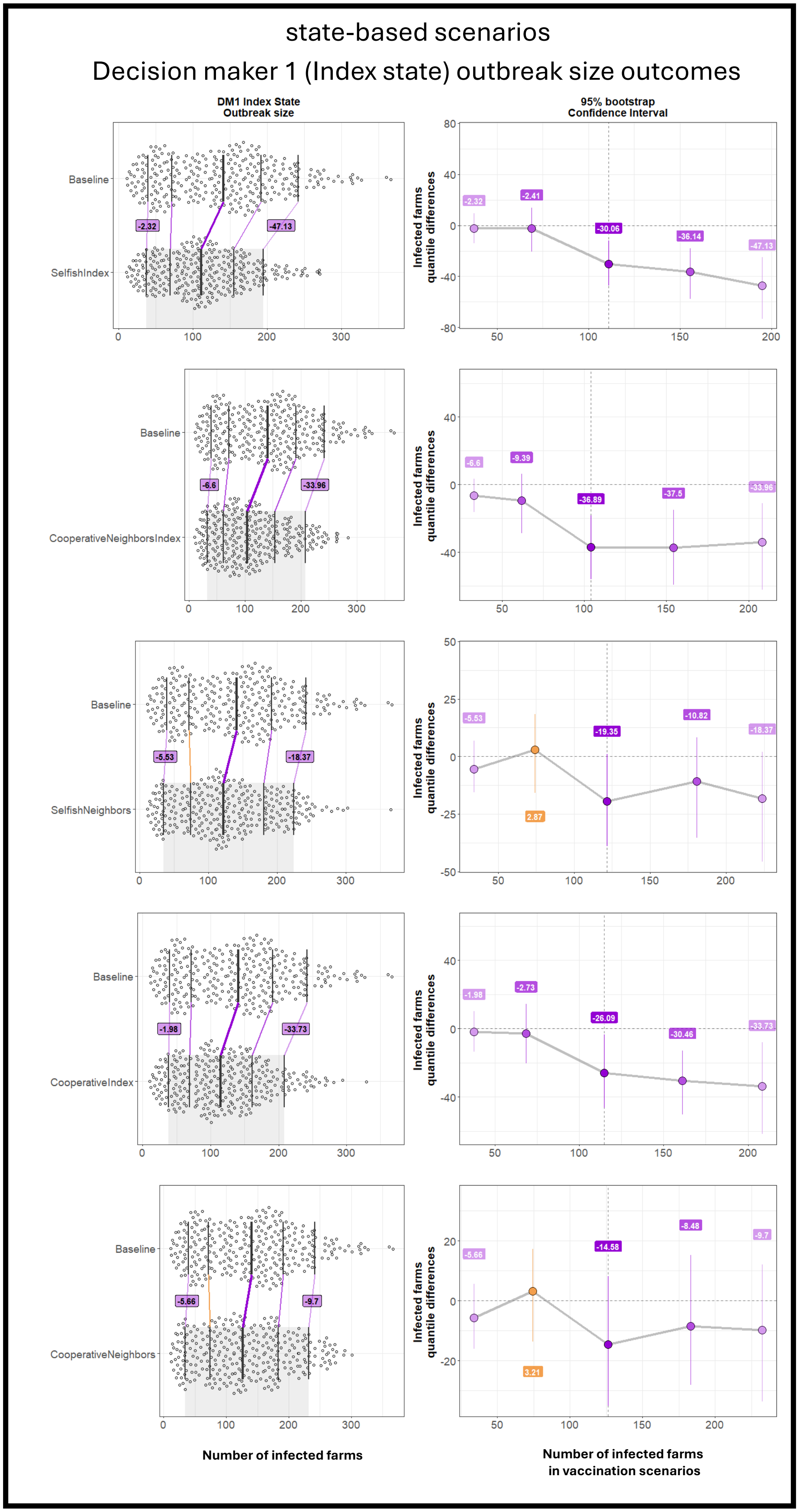

Figure S7. Scatter plots with shift function and 95% bootstrap confidence interval plots comparing each state-based vaccination scenario to the baseline for the number of infected farms and decision maker 1 (DM1 Index state).

Vertical lines in the scatter plots represent the 10th, 25th, 50th, 75th, and 90th deciles. For each decile, the shift function indicates by how much one distribution needs to be shifted to match the other (Rousselet, Pernet, & Wilcox, 2017).

For example, compared to the Baseline scenario (no vaccination), the Cooperative Neighbors & Index scenario (shared vaccines) had 33.93 fewer farms infected at the 90th decile. For the 95% bootstrap confidence interval plot, when the confidence interval does not include zero for the difference in the number of infected farms at each decile, it is considered significant with an alpha threshold of 0.05.

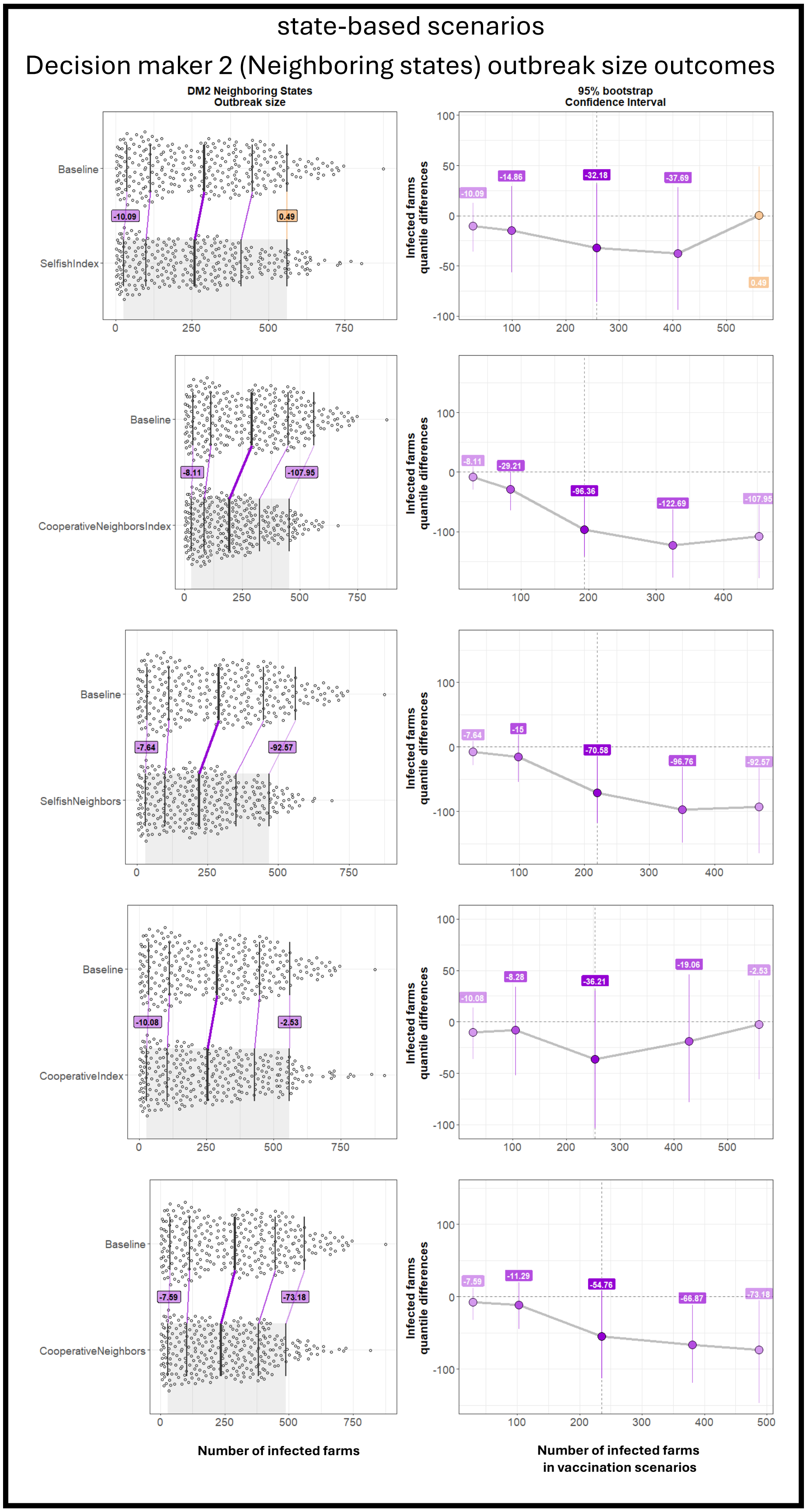

Figure S8. Scatter plots with shift function and 95% bootstrap confidence interval plots comparing each state-based vaccination scenario to the baseline for the number of infected farms and decision maker 2 (DM2 Neighboring states).

Vertical lines in the scatter plots represent the 10th, 25th, 50th, 75th, and 90th deciles. For each decile, the shift function indicates by how much one distribution needs to be shifted to match the other (Rousselet, Pernet, & Wilcox, 2017).

For example, compared to the Baseline scenario (no vaccination), the Cooperative Neighbors & Index scenario (shared vaccines) had 107.95 fewer farms infected at the 90th decile. For the 95% bootstrap confidence interval plot, when the confidence interval does not include zero for the difference in the number of infected farms at each decile, it is considered significant with an alpha threshold of 0.05.

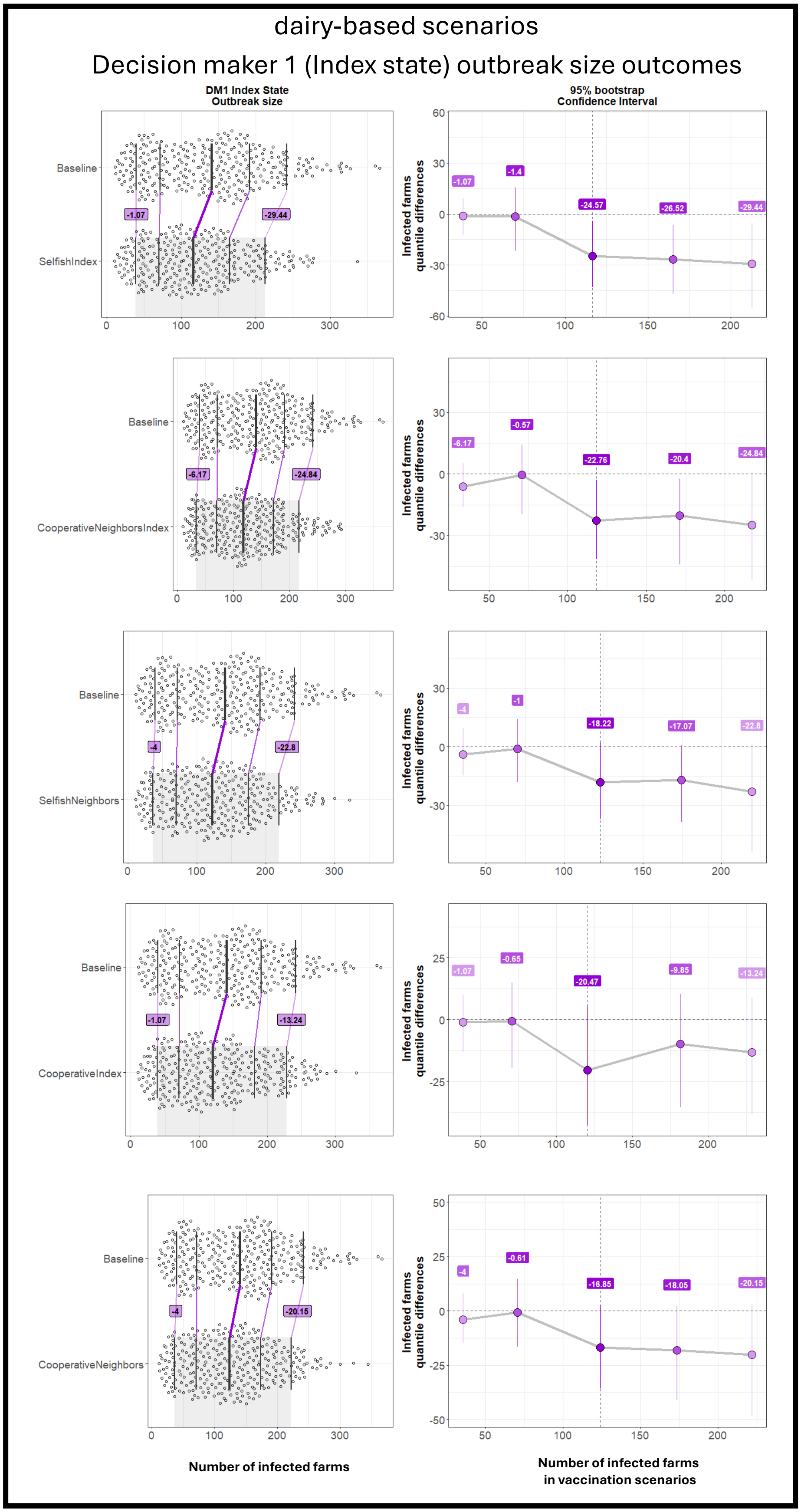

Figure S9. Scatter plots with shift function and 95% bootstrap confidence interval plots comparing each dairy-based vaccination scenario to the baseline for the number of infected farms and decision maker 1 (DM1 Index state).

Vertical lines in the scatter plots represent the 10th, 25th, 50th, 75th, and 90th deciles. For each decile, the shift function indicates by how much one distribution needs to be shifted to match the other (Rousselet, Pernet, & Wilcox, 2017).

For example, compared to the Baseline scenario (no vaccination), the Cooperative Neighbors & Index scenario (shared vaccines) had 24.84 fewer farms infected at the 90th decile. For the 95% bootstrap confidence interval plot, when the confidence interval does not include zero for the difference in the number of infected farms at each decile, it is considered significant with an alpha threshold of 0.05.

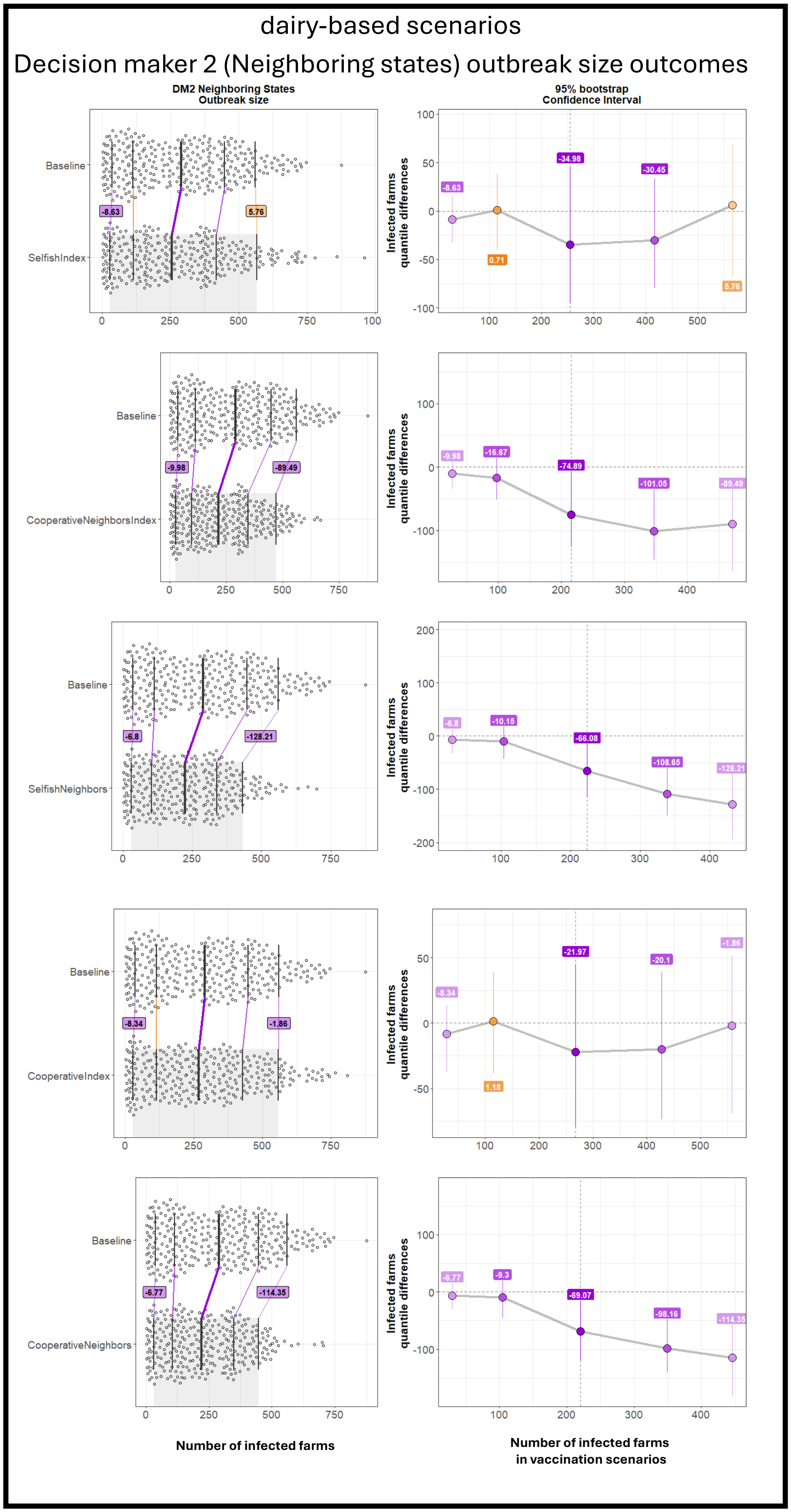

Figure S10. Scatter plots with shift function and 95% bootstrap confidence interval plots comparing each dairy-based vaccination scenario to the baseline for the number of infected farms and decision maker 2 (DM2 Neighboring states).

Vertical lines in the scatter plots represent the 10th, 25th, 50th, 75th, and 90th deciles. For each decile, the shift function indicates by how much one distribution needs to be shifted to match the other (Rousselet, Pernet, & Wilcox, 2017).

For example, compared to the Baseline scenario (no vaccination), the Cooperative Neighbors & Index scenario (shared vaccines) had 89.49 fewer farms infected at the 90th decile. For the 95% bootstrap confidence interval plot, when the confidence interval does not include zero for the difference in the number of infected farms at each decile, it is considered significant with an alpha threshold of 0.05.

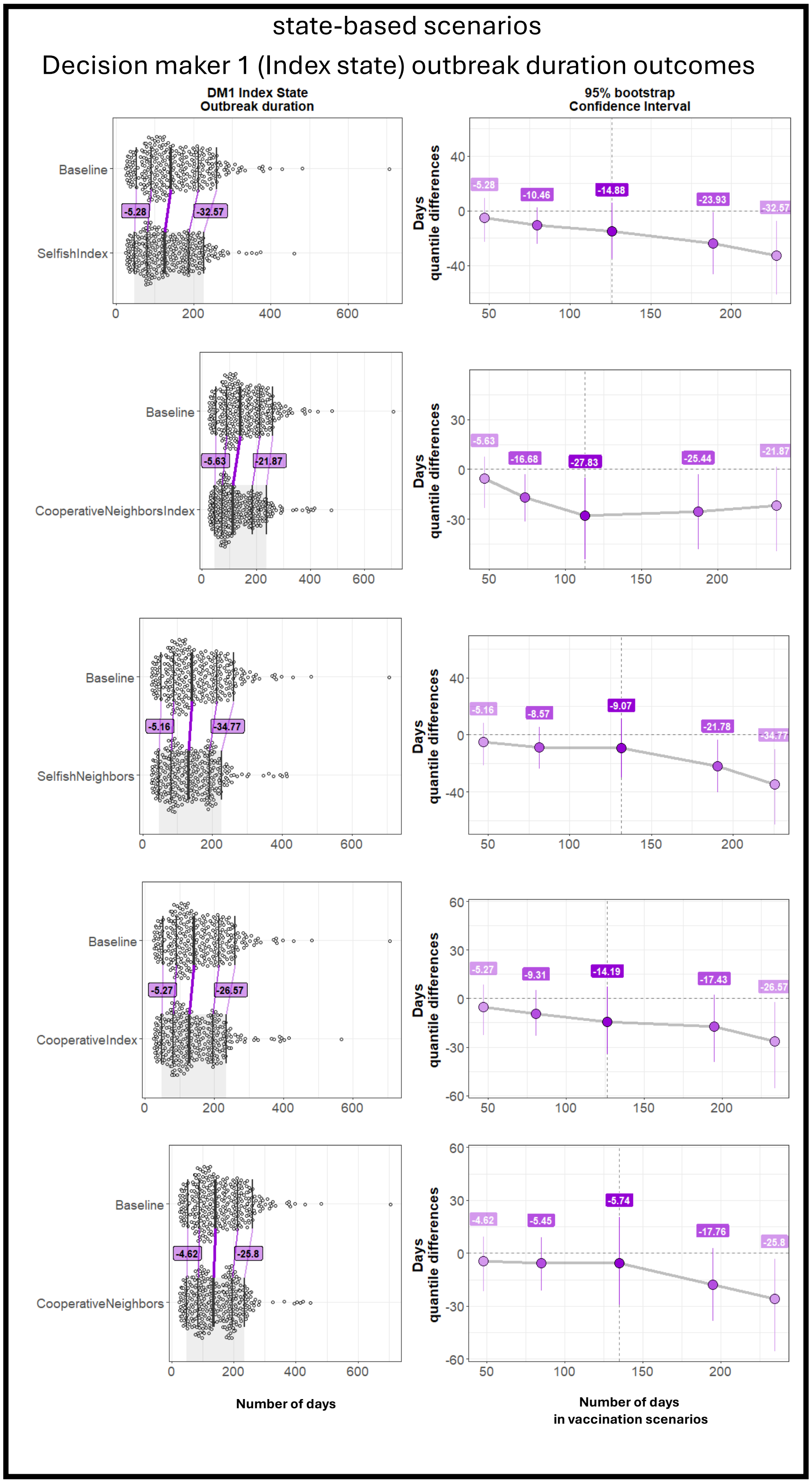

Figure S11. Scatter plots with shift function and 95% bootstrap confidence interval plots comparing each state-based vaccination scenario to the baseline for the outbreak duration in days and decision maker 1 (DM1 Index state).

Vertical lines in the scatter plots represent the 10th, 25th, 50th, 75th, and 90th deciles. For each decile, the shift function indicates by how much one distribution needs to be shifted to match the other (Rousselet, Pernet, & Wilcox, 2017).

For example, compared to the Baseline scenario (no vaccination), the Cooperative Neighbors & Index scenario (shared vaccines) resulted in 21.87 fewer days of infection at the 90th decile. For the 95% bootstrap confidence interval plot, when the confidence interval does not include zero for the difference in the number of days at each decile, it is considered significant with an alpha threshold of 0.05.

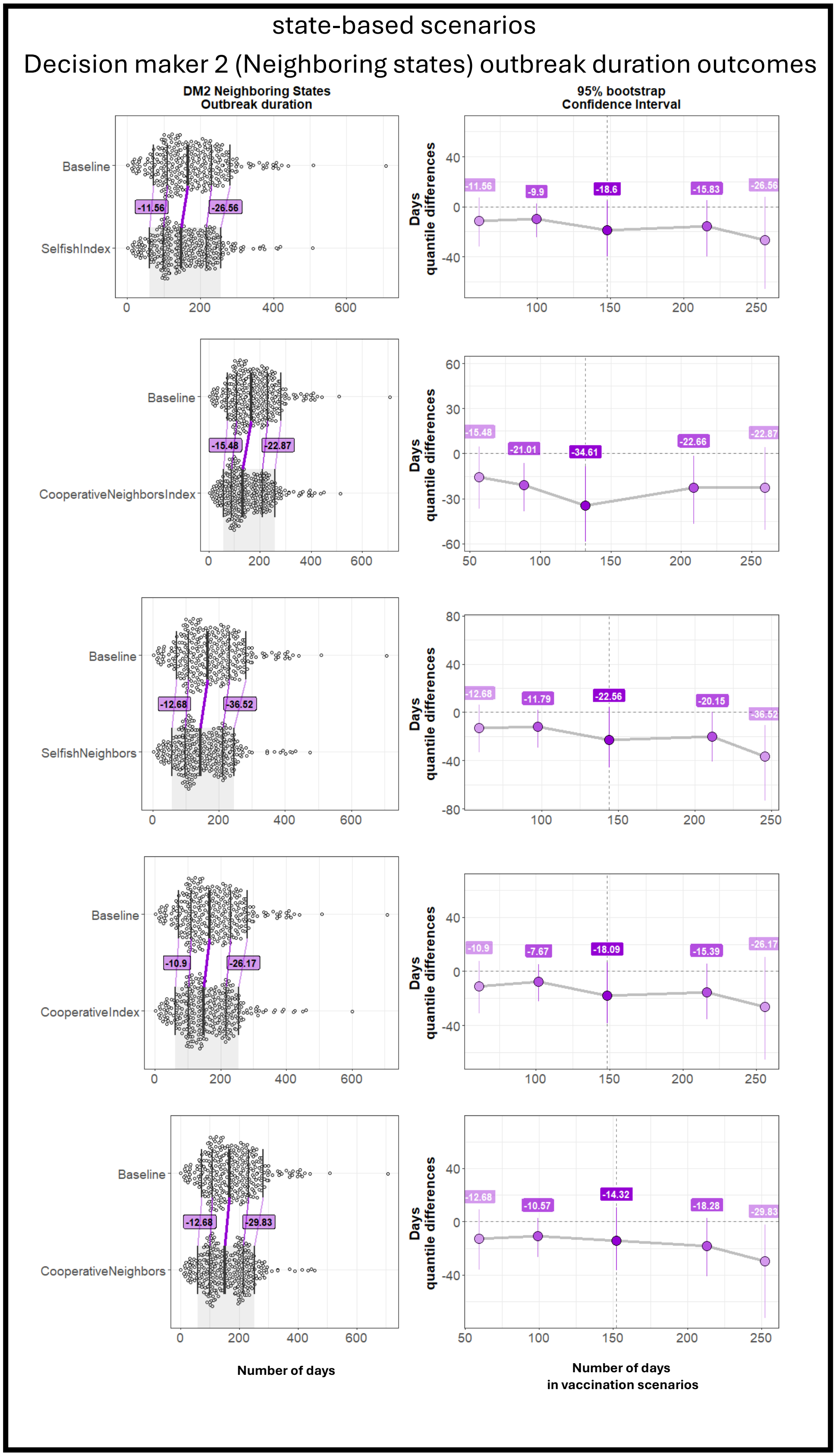

Figure S12. Scatter plots with shift function and 95% bootstrap confidence interval plots comparing each state-based vaccination scenario to the baseline for the outbreak duration in days and decision maker 2 (DM2 Neighboring states).

Vertical lines in the scatter plots represent the 10th, 25th, 50th, 75th, and 90th deciles. For each decile, the shift function indicates by how much one distribution needs to be shifted to match the other (Rousselet, Pernet, & Wilcox, 2017).

For example, compared to the Baseline scenario (no vaccination), the Cooperative Neighbors & Index scenario (shared vaccines) resulted in 22.87 fewer days of infection at the 90th decile. For the 95% bootstrap confidence interval plot, when the confidence interval does not include zero for the difference in the number of days at each decile, it is considered significant with an alpha threshold of 0.05.

dairy-based scenarios

Decision maker 1 (Index state) outbreak duration outcomes

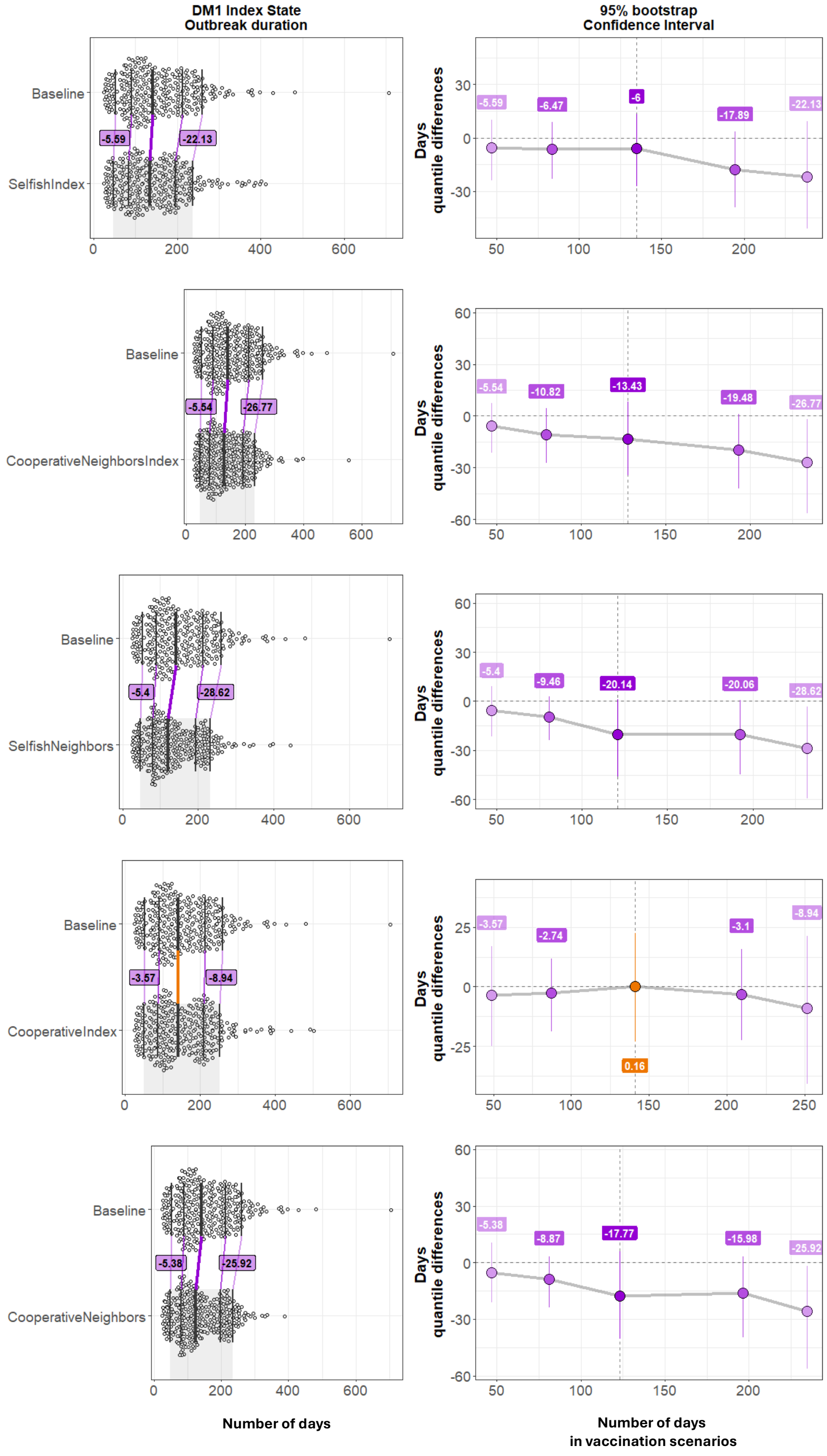

Figure S13. Scatter plots with shift function and 95% bootstrap confidence interval plots comparing each dairy-based vaccination scenario to the baseline for the outbreak duration in days and decision maker 1 (DM1 Index state).

Vertical lines in the scatter plots represent the 10th, 25th, 50th, 75th, and 90th deciles. For each decile, the shift function indicates by how much one distribution needs to be shifted to match the other (Rousselet, Pernet, & Wilcox, 2017).

For example, compared to the Baseline scenario (no vaccination), the Cooperative Neighbors & Index scenario (shared vaccines) resulted in 26.77 fewer days of infection at the 90th decile. For the 95% bootstrap confidence interval plot, when the confidence interval does not include zero for the difference in the number of days at each decile, it is considered significant with an alpha threshold of 0.05.

dairy-based scenarios

Decision maker 2 (Neighboring states) outbreak duration outcomes

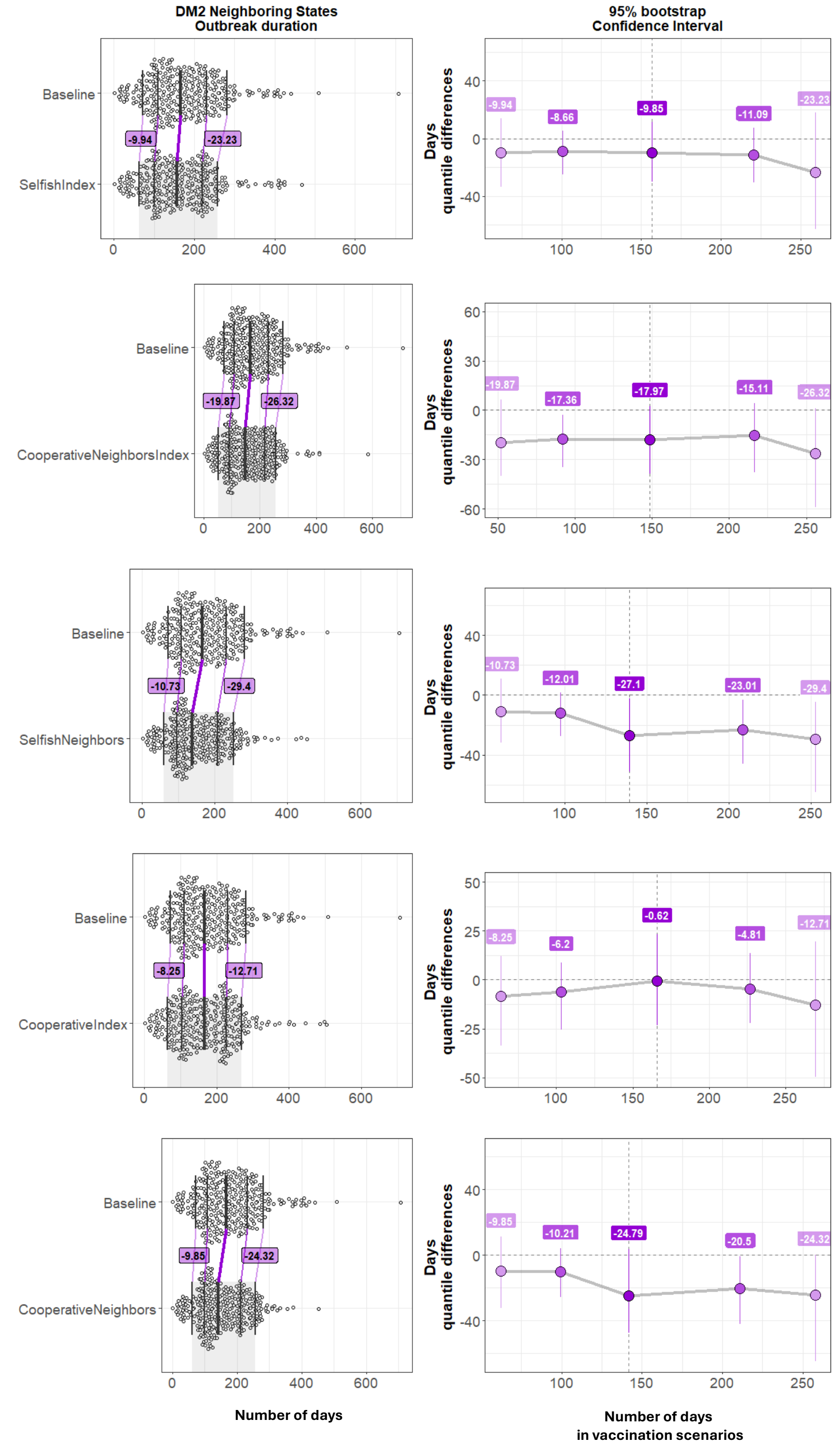

Figure S14. Scatter plots with shift function and 95% bootstrap confidence interval plots comparing each dairy-based vaccination scenario to the baseline for the outbreak duration in days and decision maker 2 (DM2 Neighboring states).

Vertical lines in the scatter plots represent the 10th, 25th, 50th, 75th, and 90th deciles. For each decile, the shift function indicates by how much one distribution needs to be shifted to match the other (Rousselet, Pernet, & Wilcox, 2017).

For example, compared to the Baseline scenario (no vaccination), the Cooperative Neighbors & Index scenario (shared vaccines) resulted in 26.32 fewer days of infection at the 90th decile. For the 95% bootstrap confidence interval plot, when the confidence interval does not include zero for the difference in the number of days at each decile, it is considered significant with an alpha threshold of 0.05.

Reference

Rousselet GA, Pernet CR, Wilcox RR. Beyond differences in means: robust graphical methods to compare two groups in neuroscience. European Journal of Neuroscience. 2017 Jul;46(2):1778-48
