# Supplementary material for: A stochastic framework to assess the optimal allocation of limited vaccine doses in foot-and-mouth disease outbreaks using game theory
Source: Front Vet Sci. 2026 Feb 9;13:1681056. doi: 10.3389/fvets.2026.1681056 (PMC12930634; doi:10.3389/fvets.2026.1681056)
Supplement: Supplementary file 4 [file Table_1.pdf]

Supplementary material TABLES: A stochastic framework to assess the optimal allocation of limited vaccine doses in foot-and-mouth disease outbreaks using game theory

Table S1. Descriptive statistics of the simulated national livestock population of the United States implemented in the foot-and-mouth disease scenarios.

Farm Type	Operation type	Number of Operations			Herd Size median; mean (min-max)			Number of animals		
		Large	Medium	Small	Large	Medium	Small	Large	Medium	Small
Cattle	cow-calf	48,144		678,491	350; 471 (200-13,020)		21; 36 (2-199)	22,685,472		24,408,776
	dairy	3925	1373	34,021	1294; 2522 (500-48,967)	230; 284 (106-998)	33; 70 (1-499)	9,899,477	389,928	2,389,824
	feedlot	43	416	26,126	54,865; 56,138 (35,664-100,734)	12706; 14549 (6017-34,932)	63; 229 (164-5999)	2413,920	6,052,381	6,175,776
	stocker	1871		90,623	2382; 4039 (1000-122,393)		23; 66 (2-999)	7,557,874		6,017,787
Bison	bison		2547			10; 63 (1-3623)			160,388	
Goat	goat		127,954			11; 21 (1-5252)			2,627,243	
Sheep	sheep	2922		85,013	418; 1105 (200-48,160)		12; 25 (1-199)	3,228,279		2,114,002
Swine	farrow to wean	862		322	4800; 6478 (1003-90,261)		309; 381 (100-996)	5,583,619		122,701
	farrow to feeder	124		353	4060; 7042 (1069-61,039)		193; 292 (101-978)	873,244		102,978
	farrow to finish	1480		2234	4286; 6478 (1015-167,275)		288; 349 (100-989)	9,610,581		779,554
	grower to finisher	6126		2134	4398; 5766 (1000-128,240)		466; 486 (100-999)	35,325,315		1,037,978
	nursery	1030		203	4638; 6098 (1001-58,064)		595; 557 (108-994)	6,280,779		113,043
	others	969		271	4324; 5738 (1008-174,418)		320; 388 (100-967)	5,559,984		105,081
	transitional swine		47,062			6; 11 (1-99)			508,068	
Dealer	dealer		5010			59; 59 (1- 133)			293,515	
Market	market		2647							
Processors	processors		1881							

Table S2. Descriptive statistics of the simulated Iowa livestock population of the United States implemented in the foot-and-mouth disease scenarios

Farm Type	Operation type	Number of Operations			Herd Size median; mean (min-max)			Number of animals		
		**Large**	**Medium**	**Small**	**Large**	**Medium**	**Small**	**Large**	**Medium**	**Small**
Cattle	cow-calf	1518		18,139	267; 336 (200-7415)		42; 51 (2-199)	509,631		933,503
	dairy	92	40	976	936; 2038 (500-12,797)	247; 320 (106-945)	53; 90 (1-499)	187,522	12,794	87,408
	feedlot		1	5362		6228	113; 287 (1-4890)		6228	1,540,964
	stocker	28		632	2052; 2896 (1150-6418)		34; 102 (3-979)	81,098		64,480
Bison	bison		65			21; 28 (1-96)			1838	
Goat	goat		1916			13; 27 (1-626)			51,294	
Sheep	sheep	111		2792	351; 553 (200-4403)		25; 37 (1-197)	61,374		104,350
Swine	farrow to wean	137		38	4794; 5158 (1044-14,396)		564; 530 (145-930)	706,684		20,146
	farrow to feeder	16		29	3506; 3873 (1371-9288)		313; 319 (101-934)	61,970		9253
	farrow to finish	398		523	4490; 5293 (1132-25,028)		336; 398 (100-989)	2,106,622		208,291
	grower to finisher	2515		739	4478; 5170 (1005-50,490)		554; 527 (107-999)	13,003,194		389,384
	nursery	308		92	4410; 5006 (1001-34,778)		590; 551 (109-994)	1,541,792		50,655
	others	439		46	4367; 5240 (1024-44,047)		472; 483 (101-837)	2,300,560		22,215
	transitional swine		981			9; 22 (1-95)			21,563	
Dealer	dealer		329			53; 57 (1-118)			18,598	
Market	market		221							
Processors	processors		26							

Table S3. Descriptive statistics of the simulated Minnesota livestock population of the United States implemented in the foot-and-mouth disease scenarios

**Farm Type**	**Operation type**	**Number of Operations**			**Herd Size median; mean (min-max)**			**Number of animals**		
		**Large**	**Medium**	**Small**	**Large**	**Medium**	**Small**	**Large**	**Medium**	**Small**
Cattle	cow-calf	401		13,161	254; 334 (200-2443)		21; 34 (2-199)	134,041		450,990
	dairy	187	110	2131	827; 1655 (500-18,208)	243; 303 (106-813)	75; 110 (1-499)	311,281	33,384	233,555
	feedlot			3796			58; 142 (1-4350)			537,978
	stocker	44		1462	2348; 3729 (1006-18,789)		35; 93 (4-971)	164,083		136,360
Bison	bison		97			16; 32 (1-199)			3096	
Goat	goat		1582			11; 20 (2-279)			31,426	
Sheep	sheep	111		2062	369; 510 (200-2627)		19; 34 (1-199)	56,561		70,545
Swine	farrow to wean	67		31	4066; 4788 (1054-105,25)		589; 522 (109-880)	320,816		16,168
	farrow to feeder	15		32	3263; 3478 (1501-6677)		348; 402 (101-837)	52,165		12,855
	farrow to finish	183		209	4264; 4764 (1072-13,827)		342; 403 (101-988)	871,844		84,314
	grower to finisher	963		285	4200; 4691 (1009-17,097)		402; 467 (100-984)	4,517,724		133,195
	nursery	171		37	4345; 4710 (1033-11,715)		633; 627 (209-980)	805,457		23,207
	others	151		43	4556; 4870 (1021-12,567)		633; 573 (101-967)	735,429		24,653
	transitional swine		1171			8; 16 (1-97)			18,455	
Dealer	dealer		184			57; 60 (1-119)			11,026	
Market	market		81							
Processors	processors		51							

Table S4. Descriptive statistics of the simulated Nebraska livestock population of the United States implemented in the foot-and-mouth disease scenarios

Farm Type	Operation type	Number of Operations			Herd Size median; mean (min-max)			Number of animals		
		Large	Medium	Small	Large	Medium	Small	Large	Medium	Small
Cattle	cow-calf	3904		15,439	373; 495 (200, 6104)		46; 57 (2-199)	1,930,604		886,751
	dairy	33	14	281	1501; 2046 (500-6420)	460; 461 (106-945)	3; 33 (1-499)	67,525	6450	9390
	feedlot		112	1668		8838; 10655 (6059-25,694)	167; 872 (1-5999)		1,193,310	1,454,128
	stocker	61		1582	2626; 3666 (1000-13,584)		66; 136 (3-987)	223,621		215,941
Bison	bison		89			30; 260 (1-3431)			23,174	
Goat	goat		1515			11; 17 (1-246)			25,911	
Sheep	sheep	60		1403	282; 424 (200-2831)		18; 33 (1-199)	25,451		46,997
Swine	farrow to wean	50		8	3322; 4551 (1133-21,153)		293; 415 (209-765)	227,531		3320
	farrow to feeder	5		7	3534; 7781 (2083-21,675)		131; 152 (107-229)	38,905		1065
	farrow to finish	71		170	3514; 6373 (1015-52,658)		242; 328 (101-967)	452,506		55,806
	grower to finisher	235		222	3535; 6583 (1003-54,164)		450; 490 (103-992)	1,546,889		108,796
	nursery	30		36	4684; 9121 (1151-34,140)		590; 559 (228-908)	273,624		20,132
	others	37		7	5040; 7634 (1183-29,981)		389; 462 (160-706)	282,464		3236
	transitional swine		602			8; 19 (1-99)			11,330	
Dealer	dealer		310			57; 58 (1-119)			17,930	
Market	market		129							
Processors	processors		55							

Table S5. Descriptive statistics of the simulated Wisconsin livestock population of the United States implemented in the foot-and-mouth disease scenarios

Farm Type	Operation type	Number of Operations			Herd Size median; mean (min-max)			Number of animals		
		Large	Medium	Small	Large	Medium	Small	Large	Medium	Small
Cattle	cow-calf	161		12,866	246; 353 (200-2471)		18; 27 (2-199)	56,761		348,455
	dairy	679	312	5816	976; 1373 (501-15,795)	212; 250 (106-945)	60; 98 (1-499)	932,465	77,860	568,734
	feedlot	0		2793			41; 97 (1-3269)			270,668
	stocker	43		1939	2126; 2322 (1052-6337)		29; 60 (2-995)	99,841		116,096
Bison	bison		102			21; 42 (1-450)			4246	
Goat	goat		2417			11; 23 (1-579)			54,520	
Sheep	sheep	54		2540	279; 334 (202-791)		12; 24 (1-193)	18,030		62,129
Swine	farrow to wean	8		10	1491; 3153 (1251-13,042)		174; 267 (133-620)	25,221		2667
	farrow to feeder	4		30	3064; 3184 (1804-4802)		182; 283 (102-737)	12,735		8478
	farrow to finish	44		98	1773; 2273 (1094-9159)		242; 304 (100-886)	100,008		29,758
	grower to finisher	20		38	2288; 3011 (1034-8619)		300; 355 (103-825)	60,212		13,478
	nursery	2		0	1472; 1472 (1304-1641)			2945		0
	others	6		10	2898; 4312 (1542-8761)		322; 405 (169-765)	25,871		4050
	transitional swine		2002			7; 13 (1-93)			26,160	
Dealer	dealer		165			53; 56 (1-125)			9209	
Market	market		45							
Processors	processors		57							

Table S6. The median number of doses used per day from the state-based cooperative neighbors and index allocation scenario (state-3)

State	Day								
	1	2	3	4	5	6	7	8	>9
IA	49,858	49,628	48,094	13,371	19,012	9885	11,038	6824	7978
MN	16,415	11,590	6357	8153	5627	5263	9610	7659	4499
NE	17,694	12,092	26,603	16,294	10,972	6562	17,276	15,893	10,140
WI	48,766	20,257	11,746	12,614	13,314	13,583	12,668	11,076	9824

Without prior knowledge from the state-based cooperative neighbors and index scenario (state-3), we would have assigned 6 days to allocate 284,500 doses requested by IA, assuming a capacity of 50,000 doses per animal per day. However, prior knowledge guided us in selecting the number of days required to allocate the targeted doses. For example, for scenario dairy-3, 10 days were implemented to use approximately the 284,500 doses requested in IA. There were instances in which we estimated the number of days, but we further adjusted them by assessing preliminary results of the vaccines used at the 90th percentile. Iowa (IA), Minnesota (MN), Nebraska (NE), and Wisconsin (WI).

Table S7. Descriptive statistics of the number of infected farms at first detection (14 days) from the baseline simulated foot-and-mouth disease scenario.

Scenario	*States*	*25%*	*median*	*mean*	*75%*	*90%*
Baseline	National	17	22	25	31	39
	IA_MN_NE_WI	16	21	22	28	34
	IA	12	15	16	20	24
	MN_NE_WI	3	5	6	9	12
	MN	1	2	2	3	5
	NE	1	1	2	2	3
	WI	2	4	5	6	9

Table S8. Descriptive statistics of outbreak size (number of infected premises) at the national level from simulated foot-and-mouth disease scenarios.

Scenario	*25%*	*median*	*mean*	*75%*	*90%*
baseline	326	802	997	1486	2099
state-based Selfish Index	264	687	905	1348	1960
state-based Cooperative Neighbors & Index	208	556	822	1144	2090
state-based Selfish Neighbors	292	672	880	1237	1961
state-based Cooperative Index	266	711	936	1347	2169
state-based Cooperative Neighbors	286	742	936	1420	1996
dairy-based Selfish Index	283	703	948	1338	2228
dairy-based Cooperative Neighbors & Index	246	653	879	1288	2044
dairy-based Selfish Neighbors	264	607	820	1264	1771
dairy-based Cooperative Index	282	730	986	1574	2256
dairy-based Cooperative Neighbors	264	628	831	1265	1808

Table S9. Descriptive statistics of the outbreak duration at the national level from simulated foot-and-mouth disease scenarios.

Scenario	*25%*	*median*	*mean*	*75%*	*90%*
baseline	126	198	207	268	316
state-based Selfish Index	111	179	185	246	298
state-based Cooperative Neighbors & Index	104	152	178	243	296
state-based Selfish Neighbors	113	174	185	243	300
state-based Cooperative Index	114	183	192	255	310
state-based Cooperative Neighbors	112	194	192	256	304
dairy-based Selfish Index	115	184	192	260	301
dairy-based Cooperative Neighbors & Index	105	182	187	255	302
dairy-based Selfish Neighbors	113	170	182	250	290
dairy-based Cooperative Index	120	199	199	262	305
dairy-based Cooperative Neighbors	117	168	184	252	302

Table S10. National results of Infected cattle farms by type from the baseline foot-and-mouth disease scenario.

Farm type	*Farm size*	*Percentage of iterations with at least one infection (n=300)**	*Number of Infected farms, median*	*Number of Infected farms, 90th percentile*
Cow-calf	large	99	50	140
Cow-calf	small	100	92	286
Dairy-heifer-calf	large	96	28	68
Dairy-heifer-calf	medium	99	18	45
Dairy-heifer-calf	small	97	15	36
Dairy	large	100	66	167
Dairy	small	100	53	142
Cattle Dealer	NA	99	76	224
Feedlot	large	50	4	10
Feedlot	medium	95	23	87
Feedlot	small	100	242	632
Stocker	large	94	14	40
Stocker	small	98	27	90

*The baseline scenario was simulated for 300 iterations. For instance, the percentage of simulated outbreaks with infection spread to small cow-calf farms was 100% (300/300) of the time.

Table S11. Descriptive statistics of the number of infected animals nationally from simulated foot-and-mouth disease scenarios.

Scenario	*25%*	*median*	*mean*	*75%*	*90%*
baseline	277,678	790,158	1,100,310	1,750,643	2,702,370
state-based Selfish Index	224,414	612,908	938,429	1,349,584	2,270,456
state-based Cooperative Neighbors & Index	167,064	498,156	887,840	1,201,476	2,318,678
state-based Selfish Neighbors	219,230	581,674	920,858	1,348,830	2,192,969
state-based Cooperative Index	229,339	617,266	955,702	1,465,886	2,353,449
state-based Cooperative Neighbors	225,958	641,896	982,929	1,432,971	2,480,994
dairy-based Selfish Index	229,033	676,550	1,024,857	1,450,676	2,536,560
dairy-based Cooperative Neighbors & Index	192,648	611,450	945,282	1,487,848	2,216,704
dairy-based Selfish Neighbors	222,384	564,271	868,633	1,244,724	2,156,963
dairy-based Cooperative Index	262,547	694,271	1,013,454	1,486,365	2,392,632
dairy-based Cooperative Neighbors	228,553	591,382	874,005	1,201,540	2,094,631

Table S12. Descriptive statistics of outbreak size of the state-based allocation scenarios for the individual neighboring states (DM2).

No.	Scenario	State	25%	median	mean	75%	90%
base-1	baseline	NE	16	54	71	114	168
state-2	state-based selfish index	NE	12	47	62	100	148
state-3	state-based cooperative neighbors & index	NE	11	37	54	86	136
state-4	state-based selfish neighbors	NE	12	42	59	94	146
state-5	state-based cooperative index	NE	14	43	62	99	155
state-6	state-based cooperative neighbors	NE	12	42	59	94	146
base-1	baseline	WI	60	149	145	220	265
state-2	state-based selfish index	WI	54	143	144	220	278
state-3	state-based cooperative neighbors & index	WI	45	102	109	164	207
state-4	state-based selfish neighbors	WI	57	121	121	177	228
state-5	state-based cooperative index	WI	53	140	144	220	273
state-6	state-based cooperative neighbors	WI	60	121	127	182	248
base-1	baseline	MN	24	73	85	130	187
state-2	state-based selfish index	MN	19	59	75	124	168
state-3	state-based cooperative neighbors & index	MN	17	41	61	95	147
state-4	state-based selfish neighbors	MN	21	56	68	104	151
state-5	state-based cooperative index	MN	20	63	80	124	186
state-6	state-based cooperative neighbors	MN	22	61	73	112	153

Table S13. Descriptive statistics of outbreak duration of the state-based allocation scenarios for the individual neighboring states (DM2).

No.	Scenario	State	25%	median	mean	75%	90%
base-1	baseline	NE	91	144	151	206	262
state-2	state-based selfish index	NE	77	132	136	190	227
state-3	state-based cooperative neighbors & index	NE	69	116	132	184	240
state-4	state-based selfish neighbors	NE	70	111	129	184	223
state-5	state-based cooperative index	NE	83	129	139	191	230
state-6	state-based cooperative neighbors	NE	72	128	133	191	229
base-1	baseline	WI	92	135	148	198	245
state-2	state-based selfish index	WI	92	121	135	186	226
state-3	state-based cooperative neighbors & index	WI	74	110	128	173	223
state-4	state-based selfish neighbors	WI	88	121	134	188	223
state-5	state-based cooperative index	WI	94	123	142	193	237
state-6	state-based cooperative neighbors	WI	88	123	135	190	220
base-1	baseline	MN	78	127	136	186	230
state-2	state-based selfish index	MN	73	118	125	181	221
state-3	state-based cooperative neighbors & index	MN	61	98	116	168	218
state-4	state-based selfish neighbors	MN	71	106	119	167	204
state-5	state-based cooperative index	MN	74	119	130	181	224
state-6	state-based cooperative neighbors	MN	71	112	124	178	210

Table S14. Descriptive statistics of outbreak size of the dairy-based allocation scenarios for the individual neighboring states (DM2).

No.	Scenario	State	25%	median	mean	75%	90%
base-1	baseline	NE	16	54	71	114	168
dairy-2	dairy-based selfish index	NE	15	45	67	106	166
dairy-3	dairy-based cooperative neighbors & index	NE	13	45	61	96	147
dairy-4	dairy-based selfish neighbors	NE	11	38	59	99	143
dairy-5	dairy-based cooperative index	NE	15	53	68	100	167
dairy-6	dairy-based cooperative neighbors	NE	11	38	59	99	143
base-1	baseline	WI	60	149	145	220	265
dairy-2	dairy-based selfish index	WI	59	135	144	220	274
dairy-3	dairy-based cooperative neighbors & index	WI	52	112	113	164	209
dairy-4	dairy-based selfish neighbors	WI	58	112	113	166	206
dairy-5	dairy-based cooperative index	WI	60	149	144	218	269
dairy-6	dairy-based cooperative neighbors	WI	60	113	114	165	206
base-1	baseline	MN	24	73	85	130	187
dairy-2	dairy-based selfish index	MN	23	64	81	124	179
dairy-3	dairy-based cooperative neighbors & index	MN	19	52	68	106	155
dairy-4	dairy-based selfish neighbors	MN	22	56	66	100	140
dairy-5	dairy-based cooperative index	MN	23	61	83	128	186
dairy-6	dairy-based cooperative neighbors	MN	23	56	67	104	143

Table S15. Descriptive statistics of outbreak duration of the dairy-based allocation scenarios for the individual neighboring states (DM2).

No.	Scenario	State	25%	median	mean	75%	90%
base-1	baseline	NE	91	144	151	206	262
dairy-2	dairy-based selfish index	NE	74	132	144	200	243
dairy-3	dairy-based cooperative neighbors & index	NE	75	128	134	190	235
dairy-4	dairy-based selfish neighbors	NE	74	122	131	189	227
dairy-5	dairy-based cooperative index	NE	75	137	147	207	249
dairy-6	dairy-based cooperative neighbors	NE	74	121	132	190	243
base-1	baseline	WI	92	135	148	198	245
dairy-2	dairy-based selfish index	WI	84	130	140	191	229
dairy-3	dairy-based cooperative neighbors & index	WI	78	116	132	181	234
dairy-4	dairy-based selfish neighbors	WI	85	114	130	183	223
dairy-5	dairy-based cooperative index	WI	85	135	143	202	235
dairy-6	dairy-based cooperative neighbors	WI	86	116	131	186	221
base-1	baseline	MN	78	127	136	186	230
dairy-2	dairy-based selfish index	MN	75	121	131	180	221
dairy-3	dairy-based cooperative neighbors & index	MN	64	108	119	168	221
dairy-4	dairy-based selfish neighbors	MN	70	104	119	168	215
dairy-5	dairy-based cooperative index	MN	74	120	131	188	226
dairy-6	dairy-based cooperative neighbors	MN	72	110	120	170	215

Table S16. State results of the percentage of outbreaks with infection spread to cattle farms by type from the baseline foot-and-mouth disease scenario.

Farm type	*Farm size*	*IA* Percentage of iterations (n=300)*	*MN* Percentage of iterations (n=295)*	*NE* Percentage of iterations (n=278)*	*WI* Percentage of iterations (n=299)*
Cow-calf	large	97	80	78	86
Cow-calf	small	100	91	87	94
Dairy-heifer-calf	large	81	78	55	89
Dairy-heifer-calf	medium	91	73	38	90
Dairy-heifer-calf	small	84	70	21	85
Dairy	large	100	87	72	94
Dairy	small	99	88	70	94
Dealer cattle	NA	98	83	73	94
Feedlot	large	NA	NA	NA	NA
Feedlot	medium	81	NA	96	NA
Feedlot	small	100	97	93	98
Stocker	large	46	60	55	83
Stocker	small	83	72	68	88

*The baseline scenario was simulated for 300 iterations. However, foot-and-mouth disease virus infection from the index state Iowa (IA) spread to Minnesota (MN) 98% (n=295) of the time; to Nebraska (NE) 93% (n=278) of the time, while for IA and Wisconsin (WI) was 100% (n=300) of the time. For instance, the percentage of simulated outbreaks in WI with infection spread to small cow-calf farms was 94% (281/299), while in Nebraska it was 87% (241/278) of the time.

Table S17. Vaccine doses used in the state-based and dairy-based foot-and-mouth disease simulated scenarios

Scenario	*25th percentile*	*median*	*mean*	*75th percentile*	*90th percentile*
			state-based		
Cooperative index	242,268	381,722	408,343	548,803	686,159
Selfish index	372,170	578,740	598,344	818,812	1,016,409
Cooperative neighbors & index	703,996	1,159,365	1,212,142	1,634,226	2,106,654
Cooperative neighbors	407,679	785,674	838,604	1,169,782	1,543,307
Selfish neighbors	449,286	878,862	957,485	1,360,309	1,777,469
			dairy-based		
Cooperative index	136,558	204,276	212,740	267,594	347,445
Selfish index	238,428	372,635	388,599	512,701	646,665
Cooperative neighbors & index	643,405	962,040	974,985	1,343,222	1,635,271
Cooperative neighbors	436,498	774,128	784,329	1,087,882	1,357,774
Selfish neighbors	466,078	798,361	812,349	1,122,582	1,402,021

Table S18. Percent difference of the 90th percentiles of outbreak size and duration comparing Pareto optimal and Nash equilibrium solutions.

**Rule**	**Solution concept**	**Criteria combinations**	**Decision-state**	**Scenario name**	**Vaccine used 90th percentiles**	**Outbreak size (infected farms) 90th percentiles (% difference) ***		**Outbreak duration (days) 90th percentiles (% difference) ***	
						DM1	DM2	DM1	DM2
**1**	Nash	all combinations	DS2 (AN)	state-based selfish index (state-2)	1,016,409	194	562	225	254
	Pareto	all combinations	DS6 (SS), DS9 (SA)	state-based cooperative neighbors & index (state-3)	2,106,654	207 (6.3)	453 (-24.1)	235 (4.3)	256 (0.8)
**2**	Nash	all combinations	DS7 (NA)	state-based selfish neighbors (state-4)	1,777,469	224	469	222	245
	Pareto	all combinations	DS5 (AS), DS6 (SS)	state-based cooperative neighbors & index (state-3)	2,106,654	207 (-8.2)	453 (-3.5)	235 (5.5)	256 (4.3)
**3**	Nash	all combinations	DS5 (AS), DS6 (SS), DS8 (AA), DS9 (SA)	state-based cooperative neighbors & index (state-3)	2,106,654	207	453	235	256

	Pareto	all combinations	DS5 (AS), DS6 (SS), DS8 (AA), DS9 (SA)	state-based cooperative neighbors & index (state-3)	2,106,654	207 (0)	453 (0)	235 (0)	256 (0)
	Nash	DM1 OS & DM2 OD DM1 OS & DM2 OS	DS6 (SS)	dairy-based cooperative neighbors & index (dairy-3)	1,635,271	216	466	231	253
4	Pareto	DM1 OS & DM2 OD DM1 OS & DM2 OS	DS5 (AS), DS6 (SS), DS8 (AA), DS9 (SA)	dairy-based cooperative neighbors & index (dairy-3)	1,635,271	216 (0)	466 (0)	231 (0)	253 (0)
	Nash	DM1 OD & DM2 OD	DS5 (AS), DS6 (SS)	dairy-based cooperative neighbors & index (dairy-3)	1,635,271	216	466	231	253
4	Pareto	DM1 OD & DM2 OD	DS7 (NA)	dairy-based selfish neighbors (dairy-4)	1,402,021	220 (1.8)	427 (-9.1)	227 (-1.8)	250 (-1.2)

*When both DMs vaccinate according to the Pareto optimal strategies, the percentage difference of the 90th percentiles of outbreak size and duration varies compared to the Nash equilibrium (calculation: 100-[(Nash measure*100)/Pareto measure]). For example, for Rule 1 (Index priority), the outbreak size increases by 6.3% for DM1 but decreases by 24% for DM2 when comparing Pareto to Nash strategies.

Table S19. Descriptive statistics of the number of infected animals in the four states (IA, MN, NE, and WI) from simulated foot-and-mouth disease scenarios.

Scenario	25%	median	mean	75%	90%
baseline	136,286	340,417	408,898	636,021	827,268
state-based Selfish Index	132,616	317,766	360,393	559,899	738,338
state-based Cooperative Neighbors & Index	104,342	253,908	308,887	481,786	659,661
state-based Selfish Neighbors	125,145	289,887	327,215	495,556	701,755
state-based Cooperative Index	133,636	314,850	365,711	567,421	752,728
state-based Cooperative Neighbors	129,456	307,779	354,077	527,797	750,843
dairy-based Selfish Index	130,024	321,600	377,475	582,742	790,588
dairy-based Cooperative Neighbors & Index	122,347	304,395	342,708	516,834	740,556
dairy-based Selfish Neighbors	123,504	284,440	336,843	532,709	692,966
dairy-based Cooperative Index	132,853	328,726	382,030	567,445	831,078
dairy-based Cooperative Neighbors	124,297	280,064	341,384	537,172	700,626

Tables S20A & B. An alternative visualization of Tables 6-9 to show how 'good' different scenarios are across multiple criteria at selected percentiles.

Here, we present an alternative visualization of Tables 6-9 to show how 'good' different scenarios are **across multiple criteria** at selected percentiles. Tables A & B show the median and 90th percentile, respectively. We can see Pareto optimal solutions across all four criteria, including state- and dairy-based allocations. <u>However, these tables are limited in that the decision maker must choose whether to base the analyses on minimizing the median or minimizing the risk of an extreme event, such as outbreaks at the 90th percentile. Additionally, these tables do not provide a complete decision process.</u> Therefore, we advise the reader to use this alternative visualization in combination with Table 14 to obtain a robust picture of the results (see also Supplementary material, workbook "Stochastic game process", tab "PuttingItAllTogether").

- USDA perspective
  - At the median and 90th percentile outbreaks, the state-3 scenario (shared allocation between states) is a Pareto optimal outcome (Tables A & B).
  - Rule 3 (equal prioritization) is a robust vaccine allocation policy (Table 14).
  - Rule 4 (the dairy-based allocation) may be a robust vaccine allocation policy in selected criteria combinations (Table 14).
  - At the 90th percentile outbreaks, Rule 4 (the dairy-based allocation) performs well (Table B).
- States' perspective
  - The best choice for states is sensitive to the vaccine allocation rule, and this is most clearly seen in Table 14.

Note: In Table B, dominated scenarios are easy to discern. However, among Pareto optimal outcomes, discerning how 'good' different scenarios are is challenging and potentially misleading; therefore, we suggest that the reader use Table 14 for the incorporated stochasticity in the results. For example, in Table 14, only DS6 (SS) with the dairy-3 scenario (shared allocation proportional to each state's dairy numbers) is Pareto optimal solution for Rule 4, and only for selected criteria combinations. In contrast, in Table B, Rule 4, dairy-3 and dairy-4 scenarios are a Pareto optimal outcome.

Our framework incorporated stochastic epidemiological outcomes into the game analyses; thus, the frequency of Nash equilibria and Pareto optimal solutions per **decision-state** was determined to assess robustness in the decision-making process **for each decision-state and selected criteria combination** (Table 14).

Color scheme

- minimum value in column -> this scenario is a Pareto optimal outcome
- dominated outcome (there is a different scenario that is better on all criteria)
- Pareto optimal outcome because no other row is better on all criteria

A

Scenario number	Scenario name	Median Outbreak Duration		Median Outbreak Size	
		Index	Neighbors	Index	Neighbors
base-1	baseline	140	166	141	292
state-2	state-based selfish index	126	148	110	258
state-3	state-based cooperative neighbors & index	113	132	105	190
state-4	state-based selfish neighbors	131	144	122	218
state-5	state-based cooperative index	126	149	116	254
state-6	state-based cooperative neighbors	131	152	126	240
dairy-2	dairy-based selfish index	135	157	116	250
dairy-3	dairy-based cooperative neighbors & index	127	149	118	210
dairy-4	dairy-based selfish neighbors	120	139	123	223
dairy-5	dairy-based cooperative index	140	168	120	266
dairy-6	dairy-based cooperative neighbors	122	140	124	222

B

Scenario number	Scenario name	90th percentile Outbreak Duration		90th percentile Outbreak Size	
		Index	Neighbors	Index	Neighbors
base-1	baseline	259	278	240	551
state-2	state-based selfish index	225	254	194	562
state-3	state-based cooperative neighbors & index	235	256	207	453
state-4	state-based selfish neighbors	222	245	224	469
state-5	state-based cooperative index	232	255	207	554
state-6	state-based cooperative neighbors	235	249	232	483
dairy-2	dairy-based selfish index	234	254	213	567
dairy-3	dairy-based cooperative neighbors & index	231	253	216	466
dairy-4	dairy-based selfish neighbors	227	250	220	427
dairy-5	dairy-based cooperative index	251	268	230	563
dairy-6	dairy-based cooperative neighbors	235	258	222	440
